# Long-Term Exposure to Fine Particulate Matter and the Deterioration of Estimated Glomerular Filtration Rate: A Cohort Study in Patients With Pre-End-Stage Renal Disease

**DOI:** 10.3389/fpubh.2022.858655

**Published:** 2022-04-08

**Authors:** Yu-Hsien Wu, Chih-Da Wu, Mu-Chi Chung, Cheng-Hsu Chen, Laing-You Wu, Chi-Jung Chung, Hui-Tsung Hsu

**Affiliations:** ^1^School of Medicine, China Medical University, Taichung, Taiwan; ^2^Department of Geomatics, National Cheng Kung University, Tainan, Taiwan; ^3^Adjunct Associate Research Fellow, National Institute of Environmental Health Sciences, National Health Research Institutes, Miaoli, Taiwan; ^4^Division of Nephrology, Department of Medicine, Taichung Veterans General Hospital, Taichung, Taiwan; ^5^Ph.D. Program in Translational Medicine, National Chung Hsing University, Taichung, Taiwan; ^6^Rong Hsing Research Center for Translational Medicine, National Chung Hsing University, Taichung, Taiwan; ^7^Department of Public Health, China Medical University, Taichung, Taiwan; ^8^Department of Medical Research, China Medical University Hospital, Taichung, Taiwan

**Keywords:** PM_2.5_, NO_2_, land-use regression model, eGFR, pre-ESRD

## Abstract

Limited literature has explored the effect of air pollutants on chronic kidney disease (CKD) progression, especially for patients with pre-end-stage renal disease (pre-ESRD). In this study, we reported the linear and nonlinear relationships of air pollutants of particles with diameter <2.5 μm (PM_2.5_) and nitrogen dioxide (NO_2_) with estimated glomerular filtration rate (eGFR) deterioration after adjusting for smoking status and other traditional clinical factors. This study adopted a retrospective cohort of patients with stage 3b to stage 5 CKD (*N* = 11,479) from Taichung Veterans General Hospital during January 2006 to December 2020. The eGFR deterioration was defined as a decline in eGFR > 5 ml/min/1.73 m^2^/year. Hybrid kriging/land-use regression models were used to estimate the individual exposure levels of PM_2.5_ and NO_2_. The relationships of air pollutants with eGFR deterioration were evaluated using Cox proportional hazard models. After adjusting for smoking status, baseline eGFR stages, and other traditional clinical factors, the risk of eGFR deterioration was found to increase with increasing PM_2.5_ and NO_2_ level (*p* < 0.0001 and *p* = 0.041, respectively), especially for those exposed to PM_2.5_ ≥ 31.44 μg/m^3^ or NO_2_ ≥ 15.00 ppb. Similar results were also found in the two-pollutant models. Nonlinear dose–response relationships of eGFR deterioration were observed for concentrations of 26.11 μg/m^3^ for PM_2.5_ and 15.06 ppb for NO_2_. In conclusion, linear and nonlinear associations between PM_2.5_ and NO_2_ levels and the incidence risk of eGFR deterioration were observed in patients with pre-ESRD.

## Introduction

Particulate matter (PM) is a mixture of suspended liquid and solid particles in air. The PM has a number of components, including nitrates, sulfates, ammonium, other inorganic ions, and metals (1). Air pollutants involving particles with diameter <10 μm (PM_10_) and 2.5 μm (PM_2.5_), nitrogen dioxide (NO_2_), ozone, carbon monoxide (CO), and sulfur dioxide are common health-related concerns. Exposures to high levels of air pollutants are associated with increased risk of hypertension, cardiovascular disease, chronic kidney disease (CKD), stroke, lung cancer, and death (2–11).

The high global burden of kidney disease may be attributed to air pollution (12). *In vitro* and *in vivo* studies have shown that traffic-related diesel PM exposure can induce nephrotoxicity by promoting oxidative stress, inflammation, and DNA damage (13, 14). Residential proximity to major roadways and increased levels of PM_2.5_ in a cohort of patients with acute ischemic stroke were associated with reduced estimated glomerular filtration rate (eGFR) (15). In the Veterans Administrative Normative Aging Study cohort, long-term environmental exposure to higher concentrations of ambient fine PM in elderly patients with mean eGFR of 76.5 ml/min/1.73 m^2^ was associated with increased risk of eGFR decline (8). Two United States Veterans cohort studies demonstrated significant associations between exposure to PM, NO_2_, and CO and risk of CKD, eGFR decline, and end-stage renal disease (ESRD) (5, 6). A non-CKD Taiwanese cohort study indicated that long-term exposure to ambient PM_2.5_ was associated with increased risk of CKD development (16).

However, participants in the above studies were not representative of the population of patients with advanced CKD and eGFR <45 ml/min/1.73 m^2^. Limited studies have examined the association between PM_2.5_ and CKD progression in patients with advanced CKD. Whether PM_2.5_ has an impact on the deterioration of kidney disease in this sensitive population is a topic worthy of further study. In the present study, we aimed to evaluate the relationships of air pollutants and eGFR deterioration in patients with pre-ESRD and stage 3b to stage 5 CKD.

## Materials and Methods

### Study Participants

**Figure 1:**
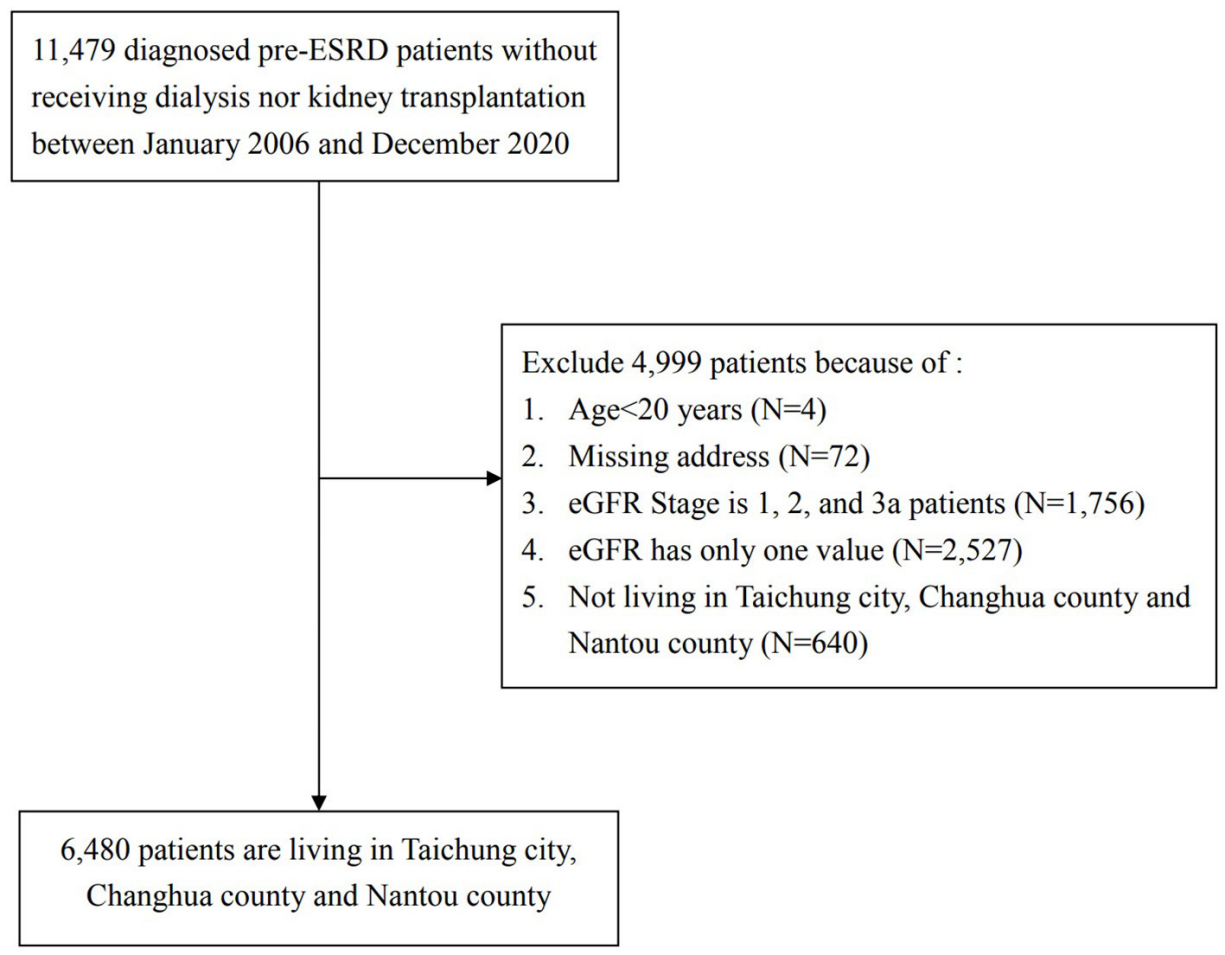
Study flow chart.

This study adopted a retrospective cohort of patients with pre-ESRD from Taichung Veterans General Hospital beginning in January 2006. All the patients joined the national integrated CKD care program, which was developed by the Taiwan Society of Nephrology and the Taiwan Health Promotion Administration, Taiwan Ministry of Health and Welfare. The detailed methods for recruitment and program construction were published by Weng et al. (17). In this study, patients with pre-ESRD (stage 3b to 5) were defined as those patients with eGFR <45 ml/min/1.73 m^2^. None of them received renal replacement therapy or kidney transplantation. The participants were included based on the following criteria: (1) age ≥ 20 years; (2) fully identified residential address; (3) recruitment between January 2006 and December 2020; (4) living in Taichung city, Changhua County, and Nantou County, Taiwan; and (5) had values of eGFR for baseline and time of deterioration. The complete study protocol is presented in Figure 1. All personal identification numbers were encrypted before being entered in the database to protect patients' privacy. This study was approved by the Research Ethics Committee of Taichung Veterans General Hospital, Taichung, Taiwan (CE19222B-1).

### Health Examinations and Collection of Covariates

All patients who participated in the study received physical and biochemistry examinations. The physical examinations included height, weight, waistline, pulse, and blood pressure. The biochemistry examination included analysis of blood glucose, hemoglobin, hematocrit value, albumin, glycated hemoglobin (HbA1c), creatinine, urine protein/creatinine ratio, total cholesterol (TC), triglycerides (TGs), high-density lipoprotein (HDL), and low-density lipoprotein (LDL). The eGFR values were calculated using Chronic Kidney Disease Epidemiology Collaboration (CKD-EPI) equation (18). Data on smoking status, alcohol consumption, history of comorbidities, and medication usage, such as anti-hypertensive, anti-diabetic, and lipid-lowering medications, were obtained through face-to-face interviews with a structured questionnaire. The status of smoking and alcohol consumption had three groups: never; former, but had quit smoking or alcohol drinking at the time of recruitment; and current.

### EGFR Deterioration

The first date of eGFR examination in clinics for all pre-ESRD patients was defined as the index date. Individual data of eGFR examination during the follow-up period were collected. The eGFR decline rate (ml/min/1.73 m^2^/year) was calculated as the difference between baseline eGFR and follow-up eGFR divided by follow-up years. The eGFR deterioration was defined as the first decline in eGFR of more than 5 ml/min/1.73 m^2^/year in the follow-up periods (19).

### PM_2.5_ and NO_2_ Estimations Through Hybrid Kriging/Land-Use Regression Model

We used Taiwan's Environmental Protection Administration air-quality monitoring data to successfully construct hybrid kriging/land-use regression (LUR) models for estimating PM_2.5_ (for 2006–2020) and NO_2_ (for 2000–2020). Related studies have used such models on health-related issues (20, 21). The hybrid kriging/LUR model included the predicted concentration level from the kriging interpolation as a variable in the LUR model and was used to estimate the levels of PM_2.5_ and NO_2_ for all study participants who provided their home address. The model determination of coefficient R^2^ and cross-validated R^2^ were, respectively, 0.85 and 0.87 for PM_2.5_, and 0.90 and 0.88 for NO_2_, thereby confirming the robustness of the developed model in predicting air pollutant variations. Based on this prediction model, we acquired the annual average concentrations of PM_2.5_ and NO_2_ for all participants. Finally, the overall average levels of PM_2.5_ and NO_2_ of all the study patients were calculated between their corresponding year of recruitment and incident CKD progression.

### Statistical Analysis

We classified all the participants into three exposure groups based on the tertile concentrations of PM_2.5_ and NO_2_, and observed the incidence of eGFR deterioration in the follow-up period. First, Chi-square tests or Kruskal–Wallis tests were used to examine the distributions of the baseline demographic, related risk factors, and the incidence of eGFR deterioration among the three exposure groups of PM_2.5_ and NO_2_. Univariate and multiple Cox proportional hazard regression models were used to evaluate the linear association between PM_2.5_ and NO_2_ levels and the incidence risk of eGFR deterioration. Calculations of person-years in the follow-up for people with and without eGFR deterioration were conducted from the index date of examination of eGFR and the first date of eGFR deterioration, death date, censored date, last date of eGFR examination, or the end of study period (31 December 2020). Furthermore, the incidence rate was defined as the incident numbers of eGFR deterioration divided by total person-years in the follow-up period. To explore the relationships of PM_2.5_ and NO_2_ with eGFR deterioration, we adjusted for age, gender, baseline eGFR stages, county of residence, education, occupation, smoking status, and sports habits. We then added PM_2.5_- and NO_2_-related risk factors, which were also associated with eGFR deterioration, into the final models – including diabetes, hypertension, gout, medication usage, and clinical examination index. In addition, we evaluated the individual effect of PM_2.5_ and NO_2_ on the risk of eGFR deterioration through two-pollutant models. We also performed combination analysis of PM_2.5_ and NO_2_ on the incidence rate risk of eGFR deterioration. We executed stratified analysis by smoking status, alcohol status, diabetes, hypertension, and CKD stage for exploring the association between PM_2.5_ and NO_2_ and risk of eGFR deterioration. Additionally, we evaluated the nonlinear relationships of air pollutants and eGFR deterioration through a distributed lag nonlinear model (dlnm package in the R program). Either b-spline or natural cubic curves used for fitting exposure–response relationships under three knots (10^th^, 50^th^, and 90^th^ percentiles of the distributions of PM_2.5_ and NO_2_) were chosen using the minimum Akaike information criterion (AIC) (22). Software SAS version 9.4 (SAS Institute, Cary, NC, USA) was used for statistical analyses. Two-tailed *p* < 0.05 was significant and we presented effect estimates with confidence intervals (CIs).

## Results

### Associations of Baseline Factors, PM_2.5_, and NO_2_ With EGFR Deterioration

According to the baseline eGFR, 2,901, 2,175, and 1,404 patients were at stages 3b, 4, and 5 of CKD in the final analysis, respectively. We separated all the study participants into three exposure groups based on the levels of PM_2.5_ and NO_2_. The comparisons of demographics, lifestyle risk factors, comorbidities, medication usage, and clinical examination index among these three exposure groups are shown in Table S1A (PM_2.5_) and 1b (NO_2_). During the follow-up duration, 3,100 patients had eGFR deterioration (about 48%), and the incidence rates were 183.11, 213.16, and 269.78 per 1,000 person-years for stages 3b, 4, and 5, respectively. The association analysis of demographics, lifestyle risk factors, comorbidities, medication usage, and clinical examination index as well as eGFR deterioration is given in Table S2. We then selected PM_2.5_- and NO_2_-related risk factors, which were also associated with eGFR deterioration, for the final multivariate models, and described these variables in Table 1. Table 1 also summarizes the information on the above important variables among the three exposure groups based on the levels of PM_2.5_ (Table 1A) and NO_2_ (Table 1B). The mean age of patients with pre-ESRD was ~75 years, and most patients were males (61.51%), lived in Taichung city (81.67%), and were non-smokers (63.93%). About 42 and 75% of the patients had comorbidities of diabetes and hypertension, respectively. Patients exposed to the highest tertile level of PM_2.5_ had high levels of systolic blood pressure, hematocrit, urea nitrogen, cholesterol, and LDL, and low hemoglobin and albumin (all *p* < 0.05). Similarly, high levels of hematocrit, urea nitrogen, cholesterol, and HbA1c were observed in those with high exposure to NO_2_.

**Table 1A:** Demographic and health characteristics of overall study cohort and according to particles with diameter <2.5 μm (PM_2.5_) level.

		**PM**_**2.5**_ **Level (μg/m**^**3**^**)**			
	**Total (*N* = 6,480)**	**Tertile 1: <25.13** **(*N* = 2,165)**	**Tertile 2: 25.13–31.44 (*N* = 2,158)**	**Tertile 3: ≥31.44** **(*N* = 2,157)**	***p* values ^**a**^**
eGFR stage 3b/ 4/ 5	2,901/ 2,175/ 1,404	1,009/ 709/ 447	1,018/ 725/ 415	874/ 741/ 542	0.0105
Incident *N* (%) of eGFR deterioration	3,100 (47.84)	870 (40.18)	985 (45.64)	1,245 (57.72)	<0.0001
Follow-up time^b^	2.32 ± 2.88	2.02 ± 2.36	3.19 ± 3.62	1.76 ± 2.26	<0.0001
Age	75.31 ± 14.84	71.24 ± 13.88	76.18 ± 14.75	78.52 ± 14.94	<0.0001
Female (%)^b^	2,494 (38.49)	878 (40.55)	802 (37.16)	814 (37.74)	0.0494
County					<0.0001
Taichung	5,292 (81.67)	1,925 (88.91)	1,835 (85.03)	1,532 (71.02)	
Nantou	537 (8.29)	129 (5.96)	155 (7.18)	253 (11.73)	
Changhua	651 (10.05)	111 (5.13)	168 (7.78)	372 (17.25)	
Education					<0.0001
Elementary school or below	3,091 (47.72)	908 (41.94)	1,070 (49.58)	1,113 (51.65)	
High school	2,125 (32.80)	821 (37.92)	668 (30.95)	636 (29.51)	
College or above	1,262 (19.48)	436 (20.14)	420 (19.46)	406 (18.84)	
Occupation					<0.0001
No	1,623 (25.06)	344 (15.90)	633 (29.33)	646 (29.98)	
Yes	1,201 (18.54)	461 (21.30)	375 (17.38)	365 (16.94)	
Other	3,653 (56.40)	1,359 (62.80)	1,150 (53.29)	1,144 (53.09)	
Smoking status					0.0002
No	4,141 (63.93)	1,456 (67.28)	1,362 (63.11)	1,323 (61.39)	
Yes	638 (9.85)	184 (8.50)	239 (11.08)	215 (9.98)	
Quit	1,698 (26.22)	524 (24.21)	557 (25.81)	617 (28.63)	
Sports habits					0.0009
No	3,182 (49.12)	995 (45.96)	1,078 (49.95)	1,109 (51.46)	
Yes	3,296 (50.88)	1,170 (54.04)	1,080 (50.05)	1,046 (48.54)	
Comorbidities					
Diabetes mellitus	2,743 (42.34)	973 (44.94)	905 (41.94)	865 (40.14)	0.0055
Hypertension	4,829 (74.54)	1,562 (72.15)	1,628 (75.44)	1,639 (76.06)	0.0065
Gout	1,255 (19.37)	373 (17.23)	465 (21.55)	417 (19.35)	0.0016
Medication usages					
EPO	2,235 (34.86)	630 (29.19)	751 (35.00)	854 (40.53)	<0.0001
Pressure pills	5,617 (87.62)	1,786 (82.76)	1,954 (91.05)	1,877 (89.08)	<0.0001
ACEI	604 (9.42)	64 (2.97)	252 (11.74)	288 (13.67)	<0.0001
CCB	3,990 (62.24)	1,280 (59.31)	1,419 (66.12)	1,291 (61.27)	<0.0001
Diuretics	2,938 (45.83)	846 (39.20)	1,045 (48.70)	1,047 (49.69)	<0.0001
Iron supplement	1,232 (19.22)	359 (16.64)	420 (19.57)	453 (21.50)	0.0003
Calcium phosphate binders	1,625 (25.35)	388 (17.98)	589 (27.45)	648 (30.75)	<0.0001
Antidiabetic drugs	2,655 (41.41)	909 (42.12)	918 (42.78)	828 (39.30)	0.0503
Biochemical examination					
SBP (mmHg)	133.31 ± 17.62	132.26 ± 17.70	133.89 ± 17.62	133.88 ± 17.49	0.0071
DBP (mmHg)	74.34 ± 11.00	74.20 ± 11.29	74.65 ± 10.91	74.14 ± 10.76	0.1529
Hemoglobin	11.19 ± 2.19	11.28 ± 2.11	11.26 ± 2.22	10.98 ± 2.28	<0.0001
Hematocrit	26.08 ± 14.71	14.71 ± 17.37	29.75 ± 12.20	32.37 ± 6.58	<0.0001
Urea Nitrogen	41.46 ± 22.15	40.58 ± 22.39	39.59 ± 20.86	44.23 ± 22.89	<0.0001
Albumin	3.99 ± 1.35	3.97 ± 0.59	4.01 ± 0.50	3.99 ± 2.22	<0.0001
Cholesterol (mg/dL)	180.11 ± 47.78	171.73 ± 46.04	181.92 ± 47.21	184.55 ± 48.82	<0.0001
LDL (mg/dL)	101.85 ± 40.61	101.65 ± 44.03	101.14 ± 35.75	103.72 ± 38.44	0.0291
HbA1c	6.78 ± 3.41	6.81 ± 5.11	6.72 ± 1.51	6.81 ± 1.61	0.141
Urine PCR	1,629.12 ± 2,627.20	1,753.81 ± 2,880.40	1,547.07 ± 2,554.20	1,558.27 ± 2,322.30	0.0738

a *p values were calculated by Chi-squares or Kruskal-Wallis test depending on categorical or continuums variables*.

b *Continuous variables were presented as mean±SD and categorical variables were presented as n (%)*.

**Table 1B:** Demographic and health characteristics of overall study cohort and according to nitrogen dioxide (NO_2_) level.

		**NO**_**2**_ **Level (ppb)**			
	**Total (*N* = 6,480)**	**Tertile 1: <12.88** **(*N* = 2,165)**	**Tertile 2: 12.88–15.00 (*N* = 2,518)**	**Tertile 3: ≥15.00** **(*N* = 2,157)**	***p* values ^**a**^**
eGFR stage 3b, 4, 5	2,901/ 2,175/ 1,404	966/ 717/ 482	1,020/ 715/ 423	915/ 743 / 499	<0.0001
Incident *N* (%) of eGFR deterioration	3,100 (47.84%)	905 (41.80)	977 (45.27)	1,218 (56.47)	<0.0001
Follow-up time	2.32 ± 2.88	2.04 ± 2.55	2.86 ± 3.27	2.06 ± 2.70	<0.0001
Age^b^	75.31 ± 14.84	72.22 ± 14.21	74.85 ± 14.70	78.86 ± 14.86	<0.0001
Female (%)^b^	2,494 (38.49)	852 (39.35)	868 (40.22)	774 (35.88)	
County					<0.0001
Taichung	5,292 (81.67)	1,522 (70.30)	1,831 (84.85)	1,939 (89.89)	
Nantou	537 (8.29)	366 (16.91)	98 (4.54)	73 (3.38)	
Changhua	651 (10.05)	277 (12.79)	229 (10.61)	145 (6.72)	
Education					<0.0001
Elementary school or below	3,091 (47.72)	1,054 (48.68)	960 (44.49)	1,077 (49.98)	
High school	2,125 (32.80)	762 (35.20)	741 (34.34)	622 (28.86)	
College or above	1,262 (19.48)	349 (16.12)	457 (21.18)	456 (21.16)	
Occupation					<0.0001
No	1,623 (25.06)	433 (20.01)	540 (25.02)	650 (30.16)	
Yes	1,201 (18.54)	488 (22.55)	408 (18.91)	305 (14.15)	
Other	3,653 (56.40)	1,243 (57.44)	1,210 (56.07)	1,200 (55.68)	
Smoking status					0.0505
No	4,141 (63.93)	1,394 (64.42)	1,415 (65.57)	1,332 (61.81)	
Yes	638 (9.85)	195 (9.01)	215 (9.96)	228 (10.58)	
Quit	1,698 (26.22)	575 (26.57)	528 (24.47)	595 (27.61)	
Sports habits					0.9571
No	3,182 (49.12)	1,069 (49.38)	1,058 (49.03)	1,055 (48.96)	
Yes	3,296 (50.88)	1,096 (50.62)	1,100 (50.97)	1,100 (51.04)	
Comorbidities					
Diabetes mellitus	2,743 (42.34)	944 (43.60)	924 (42.82)	875 (40.60)	0.1178
Hypertension	4,829 (74.54)	1,542 (71.22)	1,625 (75.30)	1,662 (77.12)	<0.0001
Gout	1,255 (19.37)	412 (19.03)	421 (19.51)	422 (19.58)	0.8829
Medication usages					
EPO	2,235 (34.86)	675 (31.34)	755 (35.17)	805 (38.15)	<0.0001
Pressure pills	5,617 (87.62)	1,790 (83.10)	1,925 (89.66)	1,902 (90.14)	<0.0001
ACEI	604 (9.42)	111 (5.15)	188 (8.76)	305 (14.45)	<0.0001
CCB	3,990 (62.24)	1,299 (60.31)	1,370 (63.81)	1,321 (62.61)	0.0551
Diuretics	2,938 (45.83)	872 (40.48)	1,028 (47.88)	1,038 (49.19)	<0.0001
Iron supplement	1,232 (19.22)	365 (16.95)	424 (19.75%)	443 (21.00)	0.0027
Calcium phosphate binders	1,625 (25.35)	434 (20.15)	565 (26.32)	626 (29.67)	<0.0001
Antidiabetic drugs	2,655 (41.41)	900 (41.78)	909 (42.34)	846 (40.09)	0.3027
Biochemical examination					
SBP (mmHg)	133.31 ± 17.62	132.93 ± 17.95	133.40 ± 17.42	133.64 ± 17.48	0.6111
DBP (mmHg)	74.34 ± 11.00	74.13 ± 11.28	74.88 ± 10.89	73.97 ± 10.79	0.0309
Hemoglobin	11.19 ± 2.19	11.22 ± 2.20	11.24 ± 2.10	11.09 ± 2.30	0.0534
Hematocrit	26.08 ± 14.71	19.59 ± 17.08	25.73 ± 15.05	32.29 ± 7.87	<0.0001
Urea Nitrogen	41.46 ± 22.15	41.31 ± 22.87	40.34 ± 21.42	42.74 ± 22.07	<0.0001
Albumin	3.99 ± 1.35	4.02 ± 2.21	3.98 ± 0.53	3.96 ± 0.51	0.0059
Cholesterol (mg/dL)	180.11 ± 47.78	174.27 ± 47.92	179.89 ± 47.28	184.94 ± 47.63	<0.0001
HbA1c	6.78 ± 3.41	6.79 ± 4.90	6.67 ± 1.48	6.90 ± 2.73	0.0224
Urine PCR	1,629.12 ± 2,627.20	1,729.23 ± 2,813.60	1,566.66 ± 2,633.40	1,576.52 ± 2,349.70	0.0015

a *p values were calculated by Chi-squares or Kruskal-Wallis test depending on categorical or continuums variables*.

b *Continuous variables were presented as mean±SD and categorical variables were presented as n (%)*.

### Linear and Nonlinear Relationships of PM_2.5_ and NO_2_ With EGFR Deterioration

**Figure 2:**
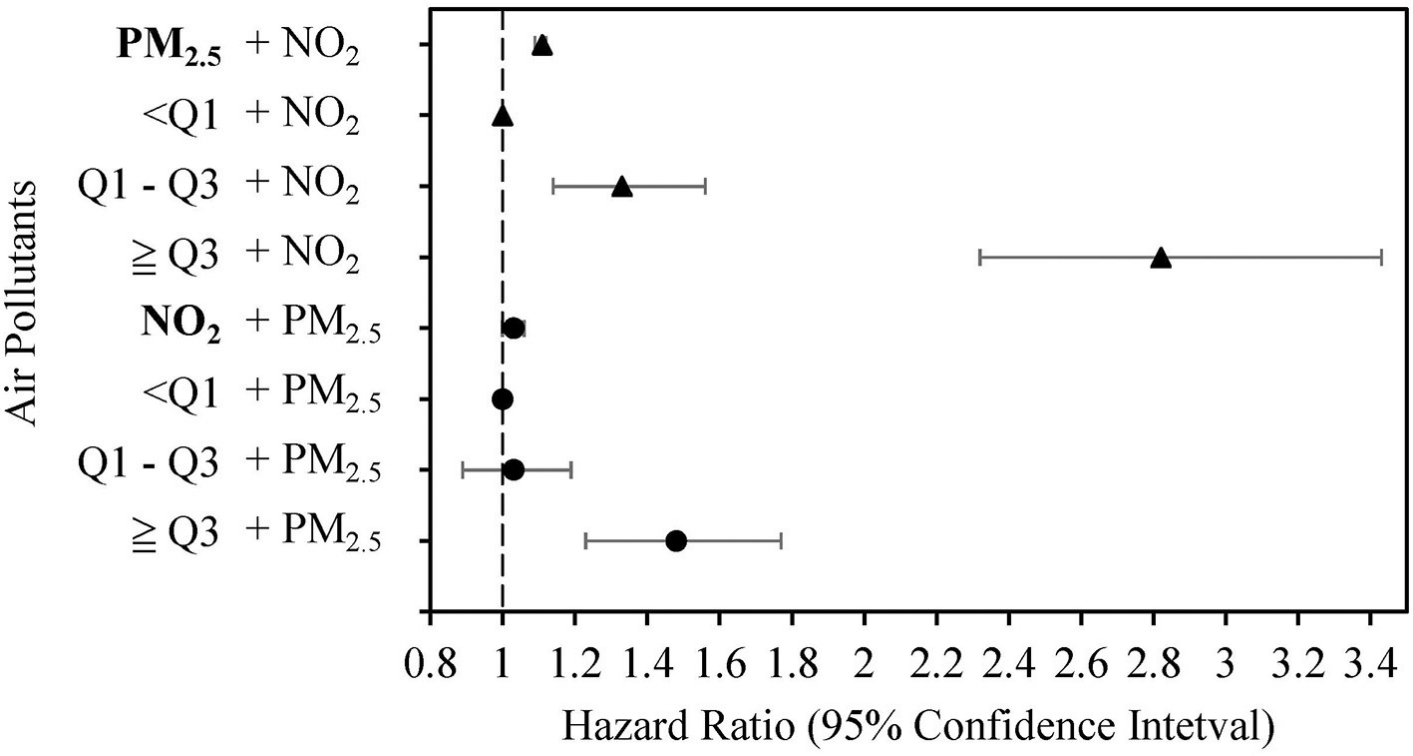
Associations of particulate matter with diameter <2.5 μm (PM_2.5_) (μg/m^3^) and nitrogen dioxide (NO_2_) (ppb) with estimated glomerular filtration rate (eGFR) deterioration in two-pollutant models after adjusting for risk factors in model 3.

Cumulative incidences of eGFR deterioration for PM_2.5_ and NO_2_ were plotted using the Kaplan-Meier method (in Figure S1). The results showed that the group with the highest PM_2.5_ and NO_2_ exposure did have a higher cumulative incidence of eGFR detoriation. We then constructed different models with adjustment for various risk factors to explore the associations of PM_2.5_ and NO_2_ with eGFR deterioration (Table 2). In the first model, we adjusted for age, gender, baseline eGFR stages, county, education, occupation, smoking status, and sports habits at baseline and found significantly increased risk of eGFR deterioration with increasing per unit of PM_2.5_ and NO_2_ [hazard ratio (HR) = 1.05 for PM_2.5_, and HR = 1.08 for NO_2_, both *p* < 0.0001]. In model 2, we additionally adjusted for comorbidities, such as diabetes, hypertension, and medication usage, which were significant factors in Table 1. Patients with PM_2.5_ ≥ 31.44 μg/m^3^ or NO_2_ ≥ 15.00 ppb had significantly increased 1.71- and 1.54-fold risks of eGFR deterioration compared with those with PM_2.5_ <25.13 μg/m^3^ or NO_2_ <12.88 ppb, respectively. Similarly, positive associations of PM_2.5_ and NO_2_ with eGFR deterioration were observed in model 3, which included the adjusted factors of model 2 and the clinical biochemistry examination. We simultaneously placed two pollutants in the same model to reduce the potential interaction of air pollutants (Figure 2). The results showed that PM_2.5_ and NO_2_ as continuous variables were still positively associated with risks of eGFR deterioration (*p* ≤ 0.0001 and *p* = 0.041, respectively), especially for those exposed to PM_2.5_ ≥ 31.44 μg/m^3^ or NO_2_ ≥ 15.00 ppb.

**Table 2:** Association analysis and combination effects between PM_2.5_ and NO_2_ as well as deterioration.

		**Single-pollutant Model**			**Two-pollutants Model**
		**Model 1**	**Model 2**	**Model 3**	
		**HR (95% CI)^**a**^**	**HR (95% CI)^**b**^**	**HR (95% CI)^**c**^**	**HR (95% CI)^**d**^**
PM_2.5_		1.05 (1.04–1.05) ^***^	1.04 (1.04–1.05) ^***^	1.12 (1.10–1.13) ^***^	1.11 (1.09–1.12) ^***^
<25.13		REF	REF	REF	REF
25.13–31.44		1.14 (1.04–1.25) ^**^	1.10 (1.00–1.21)	1.45 (1.25–1.69) ^***^	1.33 (1.14–1.56) ^***^
≥31.44		1.81 (1.65–1.98) ^***^	1.71 (1.56–1.88) ^***^	3.57 (3.02–4.23) ^***^	2.82 (2.32–3.43) ^***^
NO_2_		1.08 (1.07–1.10) ^***^	1.08 (1.06–1.09) ^***^	1.15 (1.12–1.18) ^***^	1.03 (1.00–1.06) ^*^
<12.88		REF	REF	REF	REF
12.88–15.00		1.08 (0.98–1.19)	1.04 (0.95–1.14)	1.14 (0.99–1.31)	1.03 (0.89–1.19)
≧15.00		1.60 (1.46–1.76) ^***^	1.54 (1.41–1.70) ^***^	2.38 (2.03–2.79) ^***^	1.48 (1.23–1.77) ^***^
PM_2.5_	NO_2_				
<28.78	<14.13	REF	REF	REF	p for interaction: 0.9489
<28.78	≥14.13	1.10 (0.97–1.24)	1.05 (0.93–1.20)	1.34 (1.13–1.60) ^**^	
≥28.78	<14.13	1.61 (1.42–1.82) ^***^	1.51 (1.34–1.71) ^***^	2.55 (2.09–3.11) ^***^	
≥28.78	≥14.13	1.82 (1.67–1.98) ^***^	1.73 (1.59–1.89) ^***^	3.45 (2.95–4.04) ^***^	

a *Adjustment for age, gender, baseline eGFR stages, county, education, occupation, smoking status, and sport habits*.

b *Adjustment for age, gender, baseline eGFR stages, county, education, occupation, smoking status, sport habits, diabetes, hypertension, gout, medication usage (EPO, pressure pills, ACEI, CCB, diuretics, iron supplement, calcium phosphate binders, and antidiabetic drugs)*.

c *Adjustment for age, gender, baseline eGFR stages, county, smoking, diabetes, hypertension, above medication usage, and clinical examination index (SBP, DBP, HB, Hct, urea nitrogen, albumin, cholesterol, HbA1c, and Urine PCR)*.

d *Adjustment for age, gender, baseline eGFR stages, county, smoking, diabetes, hypertension, above medication usage, and clinical examination index as well as another air pollutant*.

* *0.01 < p < 0.05*;

** *0.001 < p < 0.01*;

*** *p < 0.001*.

**Figure 3:**
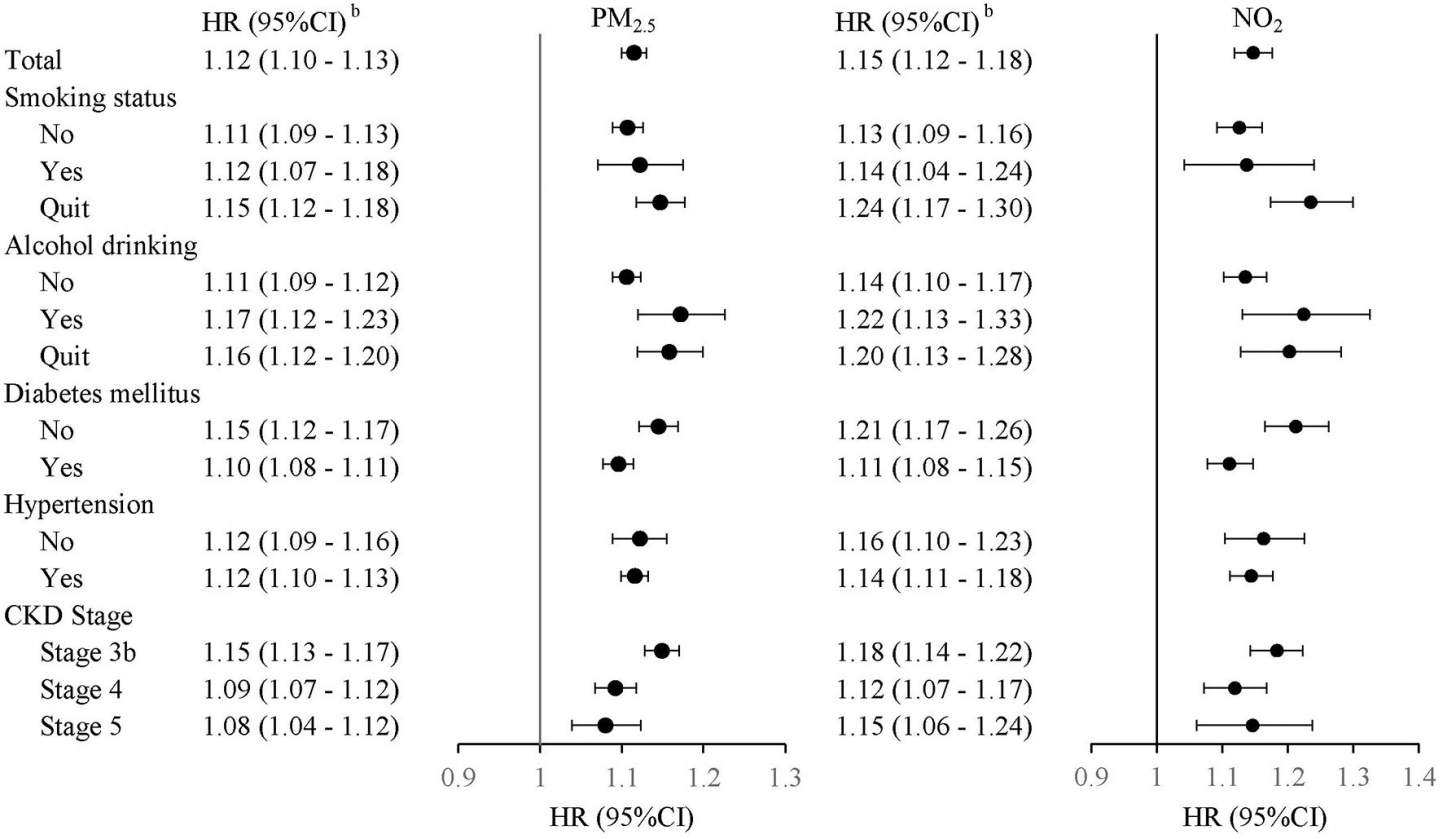
Adjusted hazard ratio (HR) with 95% confidence interval (CI) (gray area) of eGFR deterioration with PM_2.5_ and NO_2_ stratified by smoking status, alcohol status, diabetes, hypertension, and CKD stage.

We used the median values of PM_2.5_ and NO_2_ and further evaluated the joint effects of PM_2.5_ and NO_2_ on the risks of eGFR deterioration (Table 2). The results demonstrated significant 1.82-, 1.73-, and 3.45-fold risks of eGFR deterioration in models 1, 2, and 3, respectively. However, there was no obvious interaction of PM_2.5_ and NO_2_ on the risks of eGFR deterioration (*p* = 0.9489). In addition, the significant positive association between PM_2.5_, NO_2_, and eGFR deterioration was still suggested when stratifying for different factors, including smoking status, alcohol status, diabetes, hypertension, and CKD stage (Figure 3).

**Figure 4:**
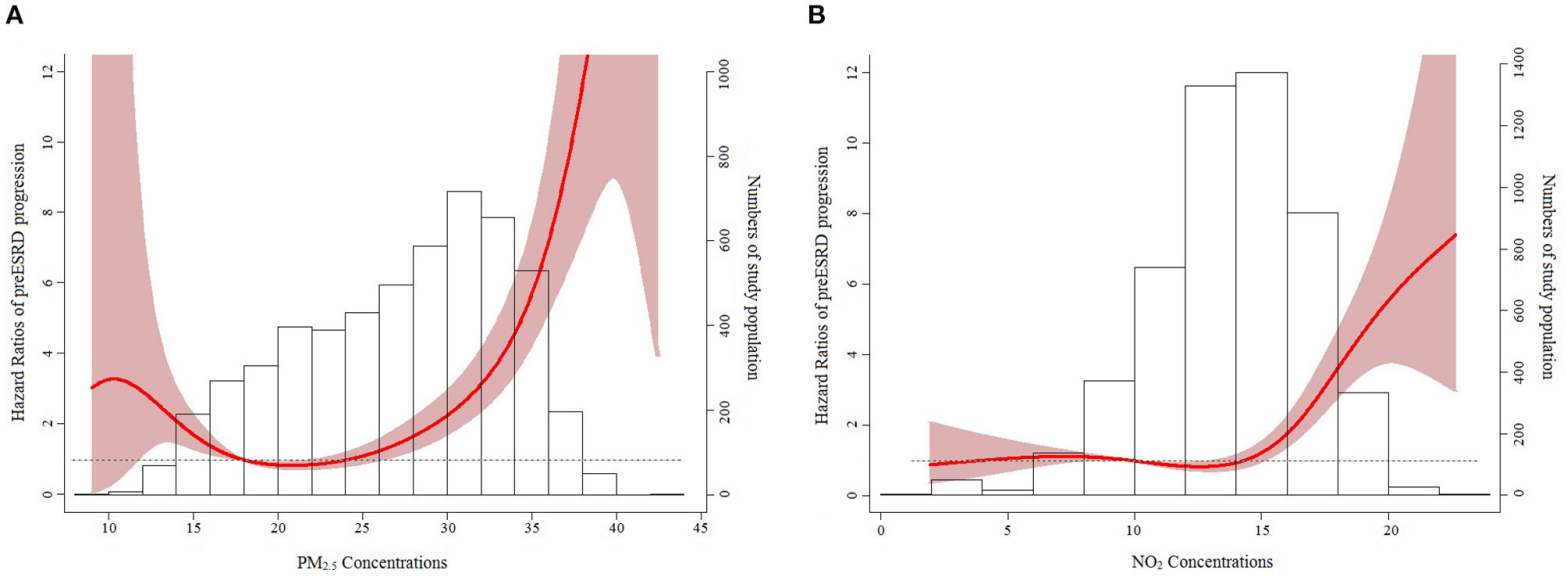
Nonlinear relationships of PM_2.5_ (μg/m^3^) **(A)** and NO_2_ (ppb) **(B)** with eGFR deterioration.

We evaluated the nonlinear effects of PM_2.5_ and NO_2_ on eGFR deterioration (Figure 4). The minimum values of AIC for PM_2.5_ and NO_2_ were respectively acquired with b-spline and natural cubic curves with their three knots of 10^th^ (as the reference value), 50^th^, and 90^th^ percentiles of the distributions of PM_2.5_ and NO_2_ (AIC = 12,959.74 and 13,048.91, respectively). Significant associations between PM_2.5_ and eGFR deterioration were found for PM_2.5_ of 11.59–17.94 μg/m^3^ as well as ≥26.11 μg/m^3^. For NO_2_, there was also a significant association with eGFR deterioration for NO_2_ ≥ 15.06 ppb.

### Sensitivity Analyses

We conducted sensitivity analyses to test the robustness of the findings (Table 3). We limited the duration of eGFR deterioration to >7, >30, and >90 days. After adjustment for other potential risk factors, significant positive relationships were found for PM_2.5_ and NO_2_ with eGFR deterioration, similar to the findings in Table 2. For the two-pollutant models, when pre-ESRD patients with eGFR deterioration within 30 or 90 days were excluded, the risk of NO_2_ per increment on eGFR deterioration disappeared. However, high NO_2_ exposure (≥15.00 ppb) still showed a positive risk of eGFR deterioration even if excluding eGFR deterioration within 30 days (HR = 1.38, *p* = 0.0009) or within 90 days (HR = 1.44, *p* = 0.0019).

**Table 3:** Sensitivity analysis for associations between PM_2.5_ and NO_2_ as well as deterioration in pre-end-stage renal disease (pre-ESRD) patients.

	**Model 1**		**Model 2**		**Model 3**	
	**HR (95% CI)** ^**a**^	***p*** **values**	**HR (95% CI)** ^**b**^	***p*** **values**	**HR (95% CI)** ^**c**^	***p*** **values**
Excluding eGFR deterioration within 7 days (deterioration: 1,854/Total population: 3,253)						
PM_2.5_	1.05 (1.04–1.05)	<0.0001	1.12 (1.10–1.13)	<0.0001	1.11 (1.09–1.12)	<0.0001
<25.13	REF		REF		REF	
25.13–31.44	1.14 (1.04–1.25)	0.0051	1.45 (1.25–1.69)	<0.0001	1.33 (1.14–1.56)	0.0004
≧31.44	1.80 (1.64–1.97)	<0.0001	3.57 (3.02–4.23)	<0.0001	2.82 (2.32–3.43)	<0.0001
NO_2_	1.08 (1.07–1.10)	<0.0001	1.15 (1.12–1.18)	<0.0001	1.03 (1.00–1.06)	0.0488
<12.88	REF		REF		REF	
12.88–15.00	1.08 (0.98–1.19)	0.1078	1.14 (0.99–1.31)	0.0668	1.03 (0.89–1.18)	0.7203
≥15.00	1.60 (1.46–1.76)	<0.0001	2.38 (2.03–2.78)	<0.0001	1.47 (1.23–1.77)	<0.0001
Excluding eGFR deterioration within 30 days (deterioration: 1,672/ Total population: 3,007)						
PM_2.5_	1.04 (1.03–1.05)	<0.0001	1.11 (1.10–1.13)	<0.0001	1.11 (1.09–1.12)	<0.0001
<25.13	REF		REF		REF	
25.13–31.44	1.08 (0.98–1.19)	0.1218	1.43 (1.23–1.67)	<0.0001	1.33 (1.14–1.57)	0.0004
≥31.44	1.65 (1.50–1.82)	<0.0001	3.42 (2.87–4.07)	<0.0001	2.80 (2.29–3.43)	<0.0001
NO_2_	1.07 (1.05–1.08)	<0.0001	1.13 (1.10–1.16)	<0.0001	1.02 (0.99–1.04)	0.3216
<12.88	REF		REF		REF	
12.88–15.00	1.05 (0.95–1.16)	0.3298	1.12 (0.97–1.29)	0.1339	1.01 (0.87–1.16)	0.9338
≥15.00	1.45 (1.32–1.60)	<0.0001	2.21 (1.88–2.60)	<0.0001	1.38 (1.14–1.67)	0.0009
Excluding eGFR deterioration within 90 days (deterioration: 1,133/ Total population: 2,359)						
PM_2.5_	1.05 (1.04–1.06)	<0.0001	1.12 (1.10–1.13)	<0.0001	1.11 (1.08–1.13)	<0.0001
<25.13	REF		REF		REF	
25.13–31.44	1.06 (0.94–1.19)	0.3282	1.38 (1.15–1.65)	0.0006	1.27 (1.05–1.54)	0.0153
≥31.44	1.77 (1.57–1.99)	<0.0001	3.52 (2.85–4.33)	<0.0001	2.80 (2.20–3.57)	<0.0001
NO_2_	1.07 (1.05–1.09)	<0.0001	1.14 (1.11–1.18)	<0.0001	1.03 (0.99–1.07)	0.123
<12.88	REF		REF		REF	
12.88–15.00	1.02 (0.91–1.15)	0.7062	1.10 (0.93–1.31)	0.2592	0.99 (0.83–1.18)	0.9194
≥15.00	1.48 (1.32–1.66)	<0.0001	2.31 (1.89–2.81)	<0.0001	1.44 (1.14–1.80)	0.0019

*Model 1: adjustment for age, gender, baseline eGFR stages, county, education, occupation, smoking status, and sport habits*.

*Model 2: adjustment for age, gender, baseline eGFR stages, county, smoking, diabetes, hypertension, medication usage, and clinical examination index*.

*Model 3: adjustment for age, gender, baseline eGFR stages, county, smoking, diabetes, hypertension, medication usage, and clinical examination index as well as another air pollutant*.

## Discussion

It has been suggested that the resolution of location-based air pollution data is frequently lower than for location-based health data. Hence, when integrating these two types of data to characterize the relationship between exposure and health, variation in the spatial correspondence between the two can be an issue. The model used to predict the exposure concentrations of PM_2.5_ and NO_2_ in this study was a kriging/LUR model. The output cell size was 50 ×50 m. The simulation model was validated using a 10-fold cross-validation approach: 90% Taiwan EPA monitoring data for model development and 10% data for validation. The R^2^ value for the kriging/LUR model was 0.85 compared with 0.66 for the conventional LUR model (21). This demonstrated that the model showed good performance in predicting the concentrations of air pollution and enhancing the quality of characterization of exposure. It improved the accuracy of generation of reliable exposure data for health risk assessment (20, 23, 24). Limited studies have explored the effect of air pollutants on CKD progression, especially for patients with pre-ESRD. Consequently, in the present work, we used a kriging/LUR model to simulate the exposure concentrations. We found linear and nonlinear relationships of PM_2.5_ and NO_2_ with eGFR deterioration after adjusting for smoking status, baseline eGFR stage, and other traditional clinical factors.

We focused on patients with pre-ESRD because patients with CKD stage 3b had a high risk of mortality and kidney outcomes compared to those with stage 3a (25). The percentage of patients with stage 3b was also lower than that of patients with stage 3a (25). In this regard, studies on patients with pre-ESRD including stages 3b, 4, and 5 are important but scarce. Previous studies showed that eGFR deterioration was consistently associated with increased risks of death (26) and ESRD (27). In the present study, we found that air pollution was a significant risk factor for eGFR deterioration in patients with pre-ESRD. This finding implies that air pollution would probably contribute to death and ESRD in these patients in the future.

Several hypotheses can explain the relationship between PM_2.5_ and eGFR deterioration. First, inhaled particles may provoke pulmonary inflammation, leading to systemic inflammation, including elevated levels of tumor necrosis factor-a (TNF-a), interleukin-6 (IL-6), plasminogen activator inhibitor-1, and oxidative stress (28, 29). Second, inhaled pollutants may induce disturbances in the autonomic nervous system, as evident in increased atherosclerotic plaque area and decreased flow-mediated dilatation (30, 31). Finally, studies also suggest that exposure to ambient air pollutants can lead to metabolic disturbances, including insulin resistance and high blood lipid concentrations, which are known risk factors for kidney diseases (32).

In Taiwan, ambient air pollution has been a severe environmental problem and has attracted considerable attention from researchers and the general public (20, 33, 34). A widespread monitoring network was implemented beginning in 1998. Air pollution data were retrieved from all 76 fixed-site air-quality monitoring stations supervised by the Taiwan Air Quality Monitoring Network. The major sources of air pollution are local traffic as well as stationary pollution, such as industrial areas, incinerators, and cremations. Approximately 80% of the study participants lived in Taichung city, within an area of about 2,200 km^2^, which included 20 industrial areas, three incinerators, two cremation sites, one large steelmaking plant, and the largest coal-fired power plant in Taiwan. Thus, extreme pollution episodes are not unusual in Taichung, especially in autumn (35). We also considered the above important land-use variables of the surrounding environment in our hybrid kriging/LUR models to estimate the levels of PM_2.5_ and NO_2_ for the residential address of each study patient (20). To reduce the potential interaction of air pollutants, we included the two pollutants in the same model. We found linear and nonlinear relationships of PM_2.5_ and NO_2_ with eGFR deterioration, especially for those exposed to the highest levels of PM_2.5_ ≥ 31.44 μg/m^3^ and NO_2_ ≥ 15.00 ppb.

One factor that cannot be ignored is that the aforementioned air pollution sources in Taichung, including thermal power plants, steel plants, and municipal waste incinerators, all utilize combustion processes and can generate incomplete combustion products. Studies have indicated that particle-bound metals play a very important role in the impact on human health (36, 37). For example, iron, manganese, lead (Pb), zinc are dominant species emitted from basic oxygen steelmaking and iron ore sinter plants (38). It is suggested that arsenic (As), selenium, mercury (Hg) are the major metals emitted with coal combustion (39, 40), and cadmium (Cd), Pb, and Hg are indicators of emissions from municipal solid waste incineration (41). Therefore, exposure to one or more of these metals is unavoidable in our study area. Moreover, metals such as As, Cd, Hg, and Pb are nephrotoxicants (42). Chronic exposure to one or more of these metals can lead to additional reductions in renal function.

Although many studies have explored the associations between air pollution and CKD incidence, this is the first study to explore eGFR deterioration of patients with pre-ESRD. Mehta et al. (8) studied long-term exposure to PM_2.5_ and decline in renal function in older adults living in the Boston area of the US. They found that for every 2.1 μg/m^3^ increase in 1-year average PM_2.5_ exposure, eGFR decreased by 1.87 ml/min/1.73 m^2^ (95% CI: −2.99 to −0.76). Another study conducted in the US also found that a 10 μg/m^3^ increase in PM_2.5_ concentration was associated with increased risk of eGFR decline of ≥ 30% in analyses considering baseline exposure (HR = 1.28; 95% CI: 1.26–1.39) (6). One study conducted in Taiwan showed that for every 8.7 μg/m^3^ increase in PM_2.5_ exposure, progression to kidney failure with replacement therapy increased by 19% (HR = 1.19; 95% CI: 1.08–1.31) (43). Our study specifically focused on pre-ESRD patients and found that a unit increase in PM_2.5_ exposure was associated with a significantly increased risk of eGFR deterioration (HR = 1.12; 95% CI: 1.10–1.13). Our results demonstrated that exposure to PM_2.5_ is a risk factor for eGFR deterioration for patients with pre-ESRD.

The strength of this study is the retrospective cohort design with at least two repeated measurements of eGFR and regular follow-up in an integrated CKD care program. However, our study has some limitations that should be carefully considered. First, the estimates of individual PM_2.5_ levels obtained using the hybrid kriging/LUR model may not reflect the actual exposure to air pollution because people may stay indoors more than 90% of the time. Second, exposure levels to air pollution were calculated as means from the period from recruitment to the date of eGFR deterioration, with an average time of about 1.5 years. A decline in renal function in such a short period of time seems unusual. We further used the various duration times from the earliest available air pollution data time to the date of eGFR deterioration (about 5.5 years) and obtained similar results (data not shown). Selecting another study design and analytical method, such as lag effect of short-term PM_2.5_ and NO_2_ exposure on eGFR deterioration, may be needed to clarify the observed relationships in future studies. Our study results indicate that exposure to high levels of PM_2.5_ and NO_2_ could be predictive risk factors for renal function decline in patients with advanced CKD. This work also supports an anti-air-pollution approach to prevent progression of advanced CKD. Additional evidence should be obtained from a variety of regions and populations to clarify the effects of PM_2.5_ and NO_2_ on renal health. Linear or nonlinear associations between the levels of PM_2.5_ and NO_2_ and the incidence risk of eGFR deterioration were observed.

## Conclusion

This cohort study on patients with advanced CKD demonstrated that exposure to high levels of PM_2.5_ and NO_2_ could promote eGFR deterioration. This finding supports the global strategy to reduce air pollution and prevent the development of ESRD.

## Data Availability Statement

The raw data supporting the conclusions of this article will be made available by the authors, without undue reservation.

## Ethics Statement

The studies involving human participants were reviewed and this study was approved by the Research Ethics Committee of Taichung Veterans General Hospital (CG20301A). All methods were performed in accordance with the relevant guidelines and regulations of Taichung Veterans General Hospital. The Ethics Committee waived the requirement of written informed consent for participation.

## Author Contributions

H-TH: had full access to all data in the study and takes responsibility for data integrity and accuracy of the data analysis, and administrative, technical, or material support. Y-HW, M-CC, C-DW, C-HC, C-JC, and H-TH: concept and design. M-CC, H-TH, L-YW, and C-JC: acquisition, analysis, or interpretation of data. M-CC, C-JC, and H-TH: manuscript drafting. M-CC and H-TH: critical revision of the manuscript for important intellectual content. L-YW: statistical analysis. All authors contributed to the article and approved the submitted version.

## Funding

This study was supported by the Ministry of Science and Technology of Taiwan under Grant No. MOST 109-2221-E-039-011-MY2 given to H-TH.

## Conflict of Interest

The authors declare that the research was conducted in the absence of any commercial or financial relationships that could be construed as a potential conflict of interest.

## Publisher's Note

All claims expressed in this article are solely those of the authors and do not necessarily represent those of their affiliated organizations, or those of the publisher, the editors and the reviewers. Any product that may be evaluated in this article, or claim that may be made by its manufacturer, is not guaranteed or endorsed by the publisher.

## Abbreviations

CKD: chronic kidney disease
ESRD: end-stage renal disease
eGFR: estimated glomerular filtration rate
PM_2._5: particles with diameter <2.5 μm
NO_2_: nitrogen dioxide.
