# Supplementary material for: Long-Term Exposure to Fine Particulate Matter and the Deterioration of Estimated Glomerular Filtration Rate: A Cohort Study in Patients With Pre-End-Stage Renal Disease
Source: Front Public Health. 2022 Apr 8;10:858655. doi: 10.3389/fpubh.2022.858655 (PMC9024125; doi:10.3389/fpubh.2022.858655)
Supplement: Supplementary file 1 [file Data_Sheet_1.docx]

| Supplement Table 1a Demographic and health characteristics of overall study cohort and according to PM_2.5_ Level | | | | | |
| --- | --- | --- | --- | --- | --- |
|  |  | PM_2.5_ Level | | |  |
|  | Total (N= 6,480) | Tertile 1 (N= 2,165) | Tertile 2 (N= 2,158) | Tertile 3 (N= 2,157) | p values ^a^ |
| eGFR stage 3b/ 4/ 5 | 2,901/ 2,175/ 1,404 | 1,009/ 709/ 447 | 1,018/ 725/ 415 | 874/ 741/ 542 |  |
| Incident N (%) of eGFR deterioration | 3,100 (47.84%) | 870 (40.18%) | 985 (45.64%) | 1,245 (57.72%) |  |
| Follow-up time | 2.32 ± 2.88 | 2.02 ± 2.36 | 3.19 ± 3.62 | 1.76 ± 2.26 | <.0001 |
| Age | 75.31 ± 14.84 | 71.24 ± 13.88 | 76.18 ± 14.75 | 78.52 ± 14.94 | <.0001 |
| Female (%) | 2,494 (38.49%) | 878 (40.55%) | 802 (37.16%) | 814 (37.74%) | 0.0494 |
| County |  |  |  |  | <.0001 |
| Taichung | 5,292 (81.67%) | 1,925 (88.91%) | 1,835 (85.03%) | 1,532 (71.02%) |  |
| Nantou | 537 (8.29%) | 129 (5.96%) | 155 (7.18%) | 253 (11.73%) |  |
| Changhua | 651 (10.05%) | 111 (5.13%) | 168 (7.78%) | 372 (17.25%) |  |
| Marriage |  |  |  |  | <.0001 |
| Married | 5,641 (87.08%) | 1,872 (86.47%) | 1,830 (84.80%) | 1,939 (89.98%) |  |
| Single | 372 (5.74%) | 114 (5.27%) | 138 (6.39%) | 120 (5.57%) |  |
| Other | 465 (7.18%) | 179 (8.27%) | 190 (8.80%) | 96 (4.45%) |  |
| Education |  |  |  |  | <.0001 |
| Elementary school or below | 3,091 (47.72%) | 908 (41.94%) | 1,070 (49.58%) | 1,113 (51.65%) |  |
| High school | 2,125 (32.80%) | 821 (37.92%) | 668 (30.95%) | 636 (29.51%) |  |
| College or above | 1,262 (19.48%) | 436 (20.14%) | 420 (19.46%) | 406 (18.84%) |  |
| Occupation |  |  |  |  | <.0001 |
| No | 1,623 (25.06%) | 344 (15.90%) | 633 (29.33%) | 646 (29.98%) |  |
| Yes | 1,201 (18.54%) | 461 (21.30%) | 375 (17.38%) | 365 (16.94%) |  |
| Other | 3,653 (56.40%) | 1,359 (62.80%) | 1,150 (53.29%) | 1,144 (53.09%) |  |
| Smoking status |  |  |  |  | 0.0002 |
| No | 4,141 (63.93%) | 1,456 (67.28%) | 1,362 (63.11%) | 1,323 (61.39%) |  |
| Yes | 638 (9.85%) | 184 (8.50%) | 239 (11.08%) | 215 (9.98%) |  |
| Quit | 1,698 (26.22%) | 524 (24.21%) | 557 (25.81%) | 617 (28.63%) |  |
| Alcohol drinking |  |  |  |  | 0.0004 |
| No | 4,829 (74.97%) | 1,661 (78.05%) | 1,615 (74.84%) | 1,553 (72.06%) |  |
| Yes | 584 (9.07%) | 170 (7.99%) | 196 (9.08%) | 218 (10.12%) |  |
| Quit | 1,028 (15.96%) | 297 (13.96%) | 347 (16.08%) | 384 (17.82%) |  |
| Betel consumption |  |  |  |  | 0.0006 |
| No | 5,929 (91.62%) | 1,942 (89.99%) | 1,976 (91.57%) | 2,011 (93.32%) |  |
| Yes | 79 (1.22%) | 24 (1.11%) | 28 (1.30%) | 27 (1.25%) |  |
| Quit | 463 (7.15%) | 192 (8.90%) | 154 (7.14%) | 117 (5.43%) |  |
| Sports habits |  |  |  |  | 0.0009 |
| No | 3,182 (49.12%) | 995 (45.96%) | 1,078 (49.95%) | 1,109 (51.46%) |  |
| Yes | 3,296 (50.88%) | 1,170 (54.04%) | 1,080 (50.05%) | 1,046 (48.54%) |  |
| Comorbidities |  |  |  |  |  |
| Diabetes mellitus | 2,743 (42.34%) | 973 (44.94%) | 905 (41.94%) | 865 (40.14%) | 0.0055 |
| Hypertension | 4,829 (74.54%) | 1,562 (72.15%) | 1,628 (75.44%) | 1,639 (76.06%) | 0.0065 |
| Gout | 1,255 (19.37%) | 373 (17.23%) | 465 (21.55%) | 417 (19.35%) | 0.0016 |
| Heart failure | 202 (3.12%) | 78 (3.60%) | 69 (3.20%) | 55 (2.55%) | 0.1345 |
| Heart disease | 246 (3.80%) | 63 (2.91%) | 109 (5.05%) | 74 (3.43%) | 0.0006 |
| Cerebrovascular disease | 253 (3.91%) | 69 (3.19%) | 99 (4.59%) | 85 (3.94%) | 0.059 |
| Cirrhosis | 310 (4.79%) | 91 (4.20%) | 113 (5.24%) | 106 (4.92%) | 0.2648 |
| Cancer | 559 (8.63%) | 194 (8.96%) | 188 (8.71%) | 177 (8.21%) | 0.6727 |
| Tuberculosis | 4 (0.06%) | 0 (0.00%) | 2 (0.09%) | 2 (0.09%) | 0.4068 |
| Hyperlipidemia | 1,479 (22.83%) | 395 (18.24%) | 613 (28.41%) | 471 (21.86%) | <.0001 |
| Retinopathy | 43 (0.66%) | 9 (0.42%) | 13 (0.60%) | 21 (0.97%) | 0.0707 |
| Anemia | 4 (0.06%) | 1 (0.05%) | 2 (0.09%) | 1 (0.05%) | 0.8514 |
| Immunity | 705 (10.88%) | 154 (7.11%) | 271 (12.56%) | 280 (12.99%) | <.0001 |
| Others | 173 (2.67%) | 58 (2.68%) | 46 (2.13%) | 69 (3.20%) | 0.0929 |
| Medication habits |  |  |  |  |  |
| Prescription drugs | 6,149 (94.92%) | 2,119 (97.88%) | 2,025 (93.84%) | 2,005 (93.04%) | <.0001 |
| Chinese medicine | 657 (10.14%) | 184 (8.50%) | 213 (9.87%) | 260 (12.06%) | 0.0005 |
| Chinese herb | 311 (4.80%) | 62 (2.86%) | 123 (5.70%) | 126 (5.85%) | <.0001 |
| Over-the-counter (OTC) drugs | 106 (1.64%) | 20 (0.92%) | 41 (1.90%) | 45 (2.09%) | 0.0053 |
| Stimulating beverage | 1 (0.02%) | 1 (0.05%) | 0 (0.00%) | 0 (0.00%) | 1 |
| Folk prescription | 92 (1.42%) | 10 (0.46%) | 40 (1.85%) | 42 (1.95%) | <.0001 |
| Health foods | 270 (4.17%) | 145 (6.70%) | 65 (3.01%) | 60 (2.78%) | <.0001 |
| Oral pain killers | 291 (4.49%) | 63 (2.91%) | 106 (4.91%) | 122 (5.66%) | <.0001 |
| Intravenous pain killers | 86 (1.33%) | 12 (0.55%) | 39 (1.81%) | 35 (1.62%) | 0.0005 |
| Unknown medication | 23 (0.36%) | 14 (0.65%) | 6 (0.28%) | 3 (0.14%) | 0.015 |
| Others | 126 (1.95%) | 42 (1.94%) | 49 (2.27%) | 35 (1.62%) | 0.3068 |
| Medication usages |  |  |  |  |  |
| EPO | 2,235 (34.86%) | 630 (29.19%) | 751 (35.00%) | 854 (40.53%) | <.0001 |
| Active vitamin D | 742 (11.57%) | 175 (8.11%) | 254 (11.84%) | 313 (14.86%) | <.0001 |
| Pressure pills | 5,617 (87.62%) | 1,786 (82.76%) | 1,954 (91.05%) | 1,877 (89.08%) | <.0001 |
| ARB | 3,323 (51.83%) | 854 (39.57%) | 1,223 (56.99%) | 1,246 (59.14%) | <.0001 |
| ACEI | 604 (9.42%) | 64 (2.97%) | 252 (11.74%) | 288 (13.67%) | <.0001 |
| CCB | 3,990 (62.24%) | 1,280 (59.31%) | 1,419 (66.12%) | 1,291 (61.27%) | <.0001 |
| Diuretics | 2,938 (45.83%) | 846 (39.20%) | 1,045 (48.70%) | 1,047 (49.69%) | <.0001 |
| Others | 3,524 (54.97%) | 1,164 (53.94%) | 1,241 (57.83%) | 1,119 (53.11%) | 0.0042 |
| Iron supplement | 1,232 (19.22%) | 359 (16.64%) | 420 (19.57%) | 453 (21.50%) | 0.0003 |
| Calcium phosphate binders | 1,625 (25.35%) | 388 (17.98%) | 589 (27.45%) | 648 (30.75%) | <.0001 |
| Antidiabetic drugs | 2,655 (41.41%) | 909 (42.12%) | 918 (42.78%) | 828 (39.30%) | 0.0503 |
| Lipid lowering drugs | 3,437 (53.61%) | 1,229 (56.95%) | 1,219 (56.80%) | 989 (46.94%) | <.0001 |
| Biochemical examination |  |  |  |  |  |
| SBP (mmHg) | 133.31 ± 17.62 | 132.26 ± 17.70 | 133.89 ± 17.62 | 133.88 ± 17.49 | 0.0071 |
| DBP (mmHg) | 74.34 ± 11.00 | 74.20 ± 11.29 | 74.65 ± 10.91 | 74.14 ± 10.76 | 0.1529 |
| Hemoglobin | 11.19 ± 2.19 | 11.28 ± 2.11 | 11.26 ± 2.22 | 10.98 ± 2.28 | <.0001 |
| Hematocrit | 26.08 ± 14.71 | 14.71 ± 17.37 | 29.75 ± 12.20 | 32.37 ± 6.58 | <.0001 |
| Urea Nitrogen | 41.46 ± 22.15 | 40.58 ± 22.39 | 39.59 ± 20.86 | 44.23 ± 22.89 | <.0001 |
| Uric acid | 7.77 ± 2.43 | 7.34 ± 2.27 | 7.93 ± 2.84 | 8.05 ± 2.05 | <.0001 |
| Albumin | 3.99 ± 1.35 | 3.97 ± 0.59 | 4.01 ± 0.50 | 3.99 ± 2.22 | <.0001 |
| Cholesterol (mg/dL) | 180.11 ± 47.78 | 171.73 ± 46.04 | 181.92 ± 47.21 | 184.55 ± 48.82 | <.0001 |
| Triglyceride (mg/dL) | 156.73 ± 114.51 | 156.62 ± 112.66 | 159.34 ± 123.91 | 154.19 ± 106.10 | 0.4959 |
| LDL (mg/dL) | 101.85 ± 40.61 | 101.65 ± 44.03 | 101.14 ± 35.75 | 103.72 ± 38.44 | 0.0291 |
| Sugar AC (mg/dL) | 118.13 ± 45.90 | 117.74 ± 46.98 | 118.32 ± 44.30 | 118.39 ± 46.35 | 0.5997 |
| HbA1c | 6.78 ± 3.41 | 6.81 ± 5.11 | 6.72 ± 1.51 | 6.81 ± 1.61 | 0.141 |
| Urine PCR | 1,629.12 ± 2,627.20 | 1,753.81 ± 2,880.40 | 1,547.07 ± 2,554.20 | 1,558.27 ± 2,322.30 | 0.0738 |
| ^a^ p values were calculated by Chi-squares or Kruskal-Wallis test depending on categorical or continuums variables. | | | | | |

| Supplement Table 1b Demographic and health characteristics of overall study cohort and according to NO_2_ Level | | | | | |
| --- | --- | --- | --- | --- | --- |
|  |  | NO_2_ Level | | |  |
|  | Total (N= 6,480) | Tertile 1 (N= 2,165) | Tertile 2 (N= 2,518) | Tertile 3 (N= 2,157) | p values ^a^ |
| eGFR stage 3b, 4, 5 | 2,901/ 2,175/ 1,404 | 966/ 717/ 482 | 1,020/ 715/ 423 | 915/ 743 / 499 |  |
| Incident N (%) of eGFR deterioration | 3,100 (47.84%) | 905 (41.80%) | 977 (45.27%) | 1,218 (56.47%) |  |
| Follow-up time | 2.32 ± 2.88 | 2.04 ± 2.55 | 2.86 ± 3.27 | 2.06 ± 2.70 | <.0001 |
| Age | 75.31 ± 14.84 | 72.22 ± 14.21 | 74.85 ± 14.70 | 78.86 ± 14.86 | <.0001 |
| Female (%) | 2,494 (38.49%) | 852 (39.35%) | 868 (40.22%) | 774 (35.88%) |  |
| County |  |  |  |  | <.0001 |
| Taichung | 5,292 (81.67%) | 1,522 (70.30%) | 1,831 (84.85%) | 1,939 (89.89%) |  |
| Nantou | 537 (8.29%) | 366 (16.91%) | 98 (4.54%) | 73 (3.38%) |  |
| Changhua | 651 (10.05%) | 277 (12.79%) | 229 (10.61%) | 145 (6.72%) |  |
| Marriage |  |  |  |  | <.0001 |
| Married | 5,641 (87.08%) | 1,885 (87.07%) | 1,819 (84.29%) | 1,937 (89.88%) |  |
| Single | 372 (5.74%) | 114 (5.27%) | 140 (6.49%) | 118 (5.48%) |  |
| Other | 465 (7.18%) | 166 (7.67%) | 199 (9.22%) | 100 (4.64%) |  |
| Education |  |  |  |  | <.0001 |
| Elementary school or below | 3,091 (47.72%) | 1,054 (48.68%) | 960 (44.49%) | 1,077 (49.98%) |  |
| High school | 2,125 (32.80%) | 762 (35.20%) | 741 (34.34%) | 622 (28.86%) |  |
| College or above | 1,262 (19.48%) | 349 (16.12%) | 457 (21.18%) | 456 (21.16%) |  |
| Occupation |  |  |  |  | <.0001 |
| No | 1,623 (25.06%) | 433 (20.01%) | 540 (25.02%) | 650 (30.16%) |  |
| Yes | 1,201 (18.54%) | 488 (22.55%) | 408 (18.91%) | 305 (14.15%) |  |
| Other | 3,653 (56.40%) | 1,243 (57.44%) | 1,210 (56.07%) | 1,200 (55.68%) |  |
| Smoking status |  |  |  |  | 0.0505 |
| No | 4,141 (63.93%) | 1,394 (64.42%) | 1,415 (65.57%) | 1,332 (61.81%) |  |
| Yes | 638 (9.85%) | 195 (9.01%) | 215 (9.96%) | 228 (10.58%) |  |
| Quit | 1,698 (26.22%) | 575 (26.57%) | 528 (24.47%) | 595 (27.61%) |  |
| Alcohol drinking |  |  |  |  | 0.0592 |
| No | 4,829 (74.97%) | 1,612 (75.75%) | 1,635 (75.76%) | 1,582 (73.41%) |  |
| Yes | 584 (9.07%) | 167 (7.85%) | 201 (9.31%) | 216 (10.02%) |  |
| Quit | 1,028 (15.96%) | 349 (16.40%) | 322 (14.92%) | 357 (16.57%) |  |
| Betel consumption |  |  |  |  | <.0001 |
| No | 5,929 (91.62%) | 1,922 (89.06%) | 1,983 (91.89%) | 2,024 (93.92%) |  |
| Yes | 79 (1.22%) | 31 (1.44%) | 24 (1.11%) | 24 (1.11%) |  |
| Quit | 463 (7.15%) | 205 (9.50%) | 151 (7.00%) | 107 (4.97%) |  |
| Sports habits |  |  |  |  | 0.9571 |
| No | 3,182 (49.12%) | 1,069 (49.38%) | 1,058 (49.03%) | 1,055 (48.96%) |  |
| Yes | 3,296 (50.88%) | 1,096 (50.62%) | 1,100 (50.97%) | 1,100 (51.04%) |  |
| Comorbidities |  |  |  |  |  |
| Diabetes mellitus | 2,743 (42.34%) | 944 (43.60%) | 924 (42.82%) | 875 (40.60%) | 0.1178 |
| Hypertension | 4,829 (74.54%) | 1,542 (71.22%) | 1,625 (75.30%) | 1,662 (77.12%) | <.0001 |
| Gout | 1,255 (19.37%) | 412 (19.03%) | 421 (19.51%) | 422 (19.58%) | 0.8829 |
| Heart failure | 202 (3.12%) | 81 (3.74%) | 72 (3.34%) | 49 (2.27%) | 0.0165 |
| Heart disease | 246 (3.80%) | 65 (3.00%) | 104 (4.82%) | 77 (3.57%) | 0.0061 |
| Cerebrovascular disease | 253 (3.91%) | 73 (3.37%) | 88 (4.08%) | 92 (4.27%) | 0.2762 |
| Cirrhosis | 310 (4.79%) | 122 (5.64%) | 92 (4.26%) | 96 (4.45%) | 0.0728 |
| Cancer | 559 (8.63%) | 204 (9.42%) | 186 (8.62%) | 169 (7.84%) | 0.1807 |
| Tuberculosis | 4 (0.06%) | 0 (0.00%) | 1 (0.05%) | 3 (0.14%) | 0.086 |
| Hyperlipidemia | 1,479 (22.83%) | 423 (19.54%) | 583 (27.02%) | 473 (21.95%) | <.0001 |
| Retinopathy | 43 (0.66%) | 11 (0.51%) | 12 (0.56%) | 20 (0.93%) | 0.1774 |
| Anemia | 705 (10.88%) | 178 (8.22%) | 261 (12.09%) | 266 (12.34%) | <.0001 |
| Immunity | 173 (2.67%) | 56 (2.59%) | 64 (2.97%) | 53 (2.46%) | 0.5622 |
| Others | 699 (10.79%) | 193 (8.91%) | 277 (12.84%) | 229 (10.63%) | 0.0002 |
| Medication habits |  |  |  |  |  |
| Prescription drugs | 6,149 (94.92%) | 2,081 (96.12%) | 2,040 (94.53%) | 2,028 (94.11%) | 0.0064 |
| Chinese medicine | 657 (10.14%) | 196 (9.05%) | 228 (10.57%) | 233 (10.81%) | 0.1163 |
| Chinese herb | 311 (4.80%) | 93 (4.30%) | 107 (4.96%) | 111 (5.15%) | 0.386 |
| Over-the-counter (OTC) drugs | 106 (1.64%) | 35 (1.62%) | 34 (1.58%) | 37 (1.72%) | 0.9316 |
| Stimulating beverage | 1 (0.02%) | 0 (0.00%) | 1 (0.05%) | 0 (0.00%) | 0.6658 |
| Folk prescription | 92 (1.42%) | 31 (1.43%) | 30 (1.39%) | 31 (1.44%) | 0.9895 |
| Health foods | 270 (4.17%) | 130 (6.00%) | 71 (3.29%) | 69 (3.20%) | <.0001 |
| Oral pain killers | 291 (4.49%) | 90 (4.16%) | 89 (4.12%) | 112 (5.20%) | 0.1538 |
| Intravenous pain killers | 86 (1.33%) | 26 (1.20%) | 22 (1.02%) | 38 (1.76%) | 0.084 |
| Unknown medication | 23 (0.36%) | 11 (0.51%) | 8 (0.37%) | 4 (0.19%) | 0.2022 |
| Others | 126 (1.95%) | 43 (1.99%) | 50 (2.32%) | 33 (1.53%) | 0.1722 |
| Medication usages |  |  |  |  |  |
| EPO | 2,235 (34.86%) | 675 (31.34%) | 755 (35.17%) | 805 (38.15%) | <.0001 |
| Active vitamin D | 742 (11.57%) | 206 (9.56%) | 240 (11.18%) | 296 (14.03%) | <.0001 |
| Pressure pills | 5,617 (87.62%) | 1,790 (83.10%) | 1,925 (89.66%) | 1,902 (90.14%) | <.0001 |
| ARB | 3,323 (51.83%) | 903 (41.92%) | 1,135 (52.86%) | 1,285 (60.90%) | <.0001 |
| ACEI | 604 (9.42%) | 111 (5.15%) | 188 (8.76%) | 305 (14.45%) | <.0001 |
| CCB | 3,990 (62.24%) | 1,299 (60.31%) | 1,370 (63.81%) | 1,321 (62.61%) | 0.0551 |
| Diuretics | 2,938 (45.83%) | 872 (40.48%) | 1,028 (47.88%) | 1,038 (49.19%) | <.0001 |
| Iron supplement | 1,232 (19.22%) | 365 (16.95%) | 424 (19.75%) | 443 (21.00%) | 0.0027 |
| Calcium phosphate binders | 1,625 (25.35%) | 434 (20.15%) | 565 (26.32%) | 626 (29.67%) | <.0001 |
| Antidiabetic drugs | 2,655 (41.41%) | 900 (41.78%) | 909 (42.34%) | 846 (40.09%) | 0.3027 |
| Lipid lowering drugs | 3,437 (53.61%) | 1,171 (54.36%) | 1,219 (56.78%) | 1,047 (49.62%) | <.0001 |
| Biochemical examination |  |  |  |  |  |
| SBP (mmHg) | 133.31 ± 17.62 | 132.93 ± 17.95 | 133.40 ± 17.42 | 133.64 ± 17.48 | 0.6111 |
| DBP (mmHg) | 74.34 ± 11.00 | 74.13 ± 11.28 | 74.88 ± 10.89 | 73.97 ± 10.79 | 0.0309 |
| Hemoglobin | 11.19 ± 2.19 | 11.22 ± 2.20 | 11.24 ± 2.10 | 11.09 ± 2.30 | 0.0534 |
| Hematocrit | 26.08 ± 14.71 | 19.59 ± 17.08 | 25.73 ± 15.05 | 32.29 ± 7.87 | <.0001 |
| Urea Nitrogen | 41.46 ± 22.15 | 41.31 ± 22.87 | 40.34 ± 21.42 | 42.74 ± 22.07 | <.0001 |
| Uric acid | 7.77 ± 2.43 | 7.55 ± 2.28 | 7.72 ± 2.17 | 8.04 ± 2.78 | <.0001 |
| Albumin | 3.99 ± 1.35 | 4.02 ± 2.21 | 3.98 ± 0.53 | 3.96 ± 0.51 | 0.0059 |
| Cholesterol (mg/dL) | 180.11 ± 47.78 | 174.27 ± 47.92 | 179.89 ± 47.28 | 184.94 ± 47.63 | <.0001 |
| Triglyceride (mg/dL) | 156.73 ± 114.51 | 154.96 ± 113.00 | 156.07 ± 108.78 | 159.21 ± 121.53 | 0.2427 |
| LDL (mg/dL) | 101.85 ± 40.61 | 101.32 ± 39.98 | 101.58 ± 44.30 | 103.53 ± 34.29 | 0.014 |
| Sugar AC (mg/dL) | 118.13 ± 45.90 | 118.16 ± 48.13 | 117.84 ± 45.58 | 118.41 ± 43.65 | 0.6465 |
| HbA1c | 6.78 ± 3.41 | 6.79 ± 4.90 | 6.67 ± 1.48 | 6.90 ± 2.73 | 0.0224 |
| Urine PCR | 1,629.12 ± 2,627.20 | 1,729.23 ± 2,813.60 | 1,566.66 ± 2,633.40 | 1,576.52 ± 2,349.70 | 0.0015 |
| ^a^ p values were calculated by Chi-squares or Kruskal-Wallis test depending on categorical or continuums variables. | | | | | |

| Supplement Table 2 Descriptive statistics for basic characteristics of study population during 2004–2018. | | | | | | |
| --- | --- | --- | --- | --- | --- | --- |
|  | Total  (N= 6,480) | eGFR deterioration  (N= 3,100) | Person-year  at risk | Incidence  density | Crude HR  (95%CI) | Adjusted HR  (95%CI)^a^ |
| Age | 75.31 ± 14.84 | 74.25 ± 15.12 |  |  | 0.99 (0.99,1.00) ^***^ | 0.99 (0.99,1.00) ^***^ |
| Sex |  |  |  |  |  |  |
| Male (%) | 3,986 (61.51%) | 1,912 (61.68%) | 9,405.90 | 203.28 | REF | REF |
| Female (%) | 2,494 (38.49%) | 1,188 (38.32%) | 5,643.78 | 210.50 | 1.01 (0.94 - 1.09) | 0.99 (0.92 - 1.06) |
| County |  |  |  |  |  |  |
| Taichung | 5,292 (81.67%) | 2,540 (81.94%) | 12,395.10 | 204.92 | 1.06 (0.94 - 1.19) | 1.08 (0.95 - 1.21) |
| Nantou | 537 (8.29%) | 266 (8.58%) | 1,091.78 | 243.64 | 1.16 (0.99 - 1.37) | 1.20 (1.01 - 1.41) ^*^ |
| Changhua | 651 (10.05%) | 294 (9.48%) | 1,562.81 | 188.12 | REF | REF |
| Marriage |  |  |  |  |  |  |
| Married | 5,641 (87.08%) | 2,680 (86.51%) | 13,190.63 | 203.18 | REF | REF |
| Single | 372 (5.74%) | 193 (6.23%) | 930.40 | 207.44 | 1.12 (0.97 - 1.30) | 0.94 (0.81 - 1.10) |
| Other | 465 (7.18%) | 225 (7.26%) | 928.04 | 242.45 | 1.01 (0.88 - 1.15) | 1.04 (0.91 - 1.19) |
| Education |  |  |  |  |  |  |
| Elementary school or below | 3,091 (47.72%) | 1,470 (47.45%) | 7,038.91 | 208.84 | REF | REF |
| High school | 2,125 (32.80%) | 1,000 (32.28%) | 4,954.25 | 201.85 | 0.97 (0.90 - 1.06) | 0.88 (0.81 - 0.96) ^**^ |
| College or above | 1,262 (19.48%) | 628 (20.27%) | 3,055.91 | 205.50 | 1.04 (0.95 - 1.14) | 0.92 (0.83 - 1.02) |
| Occupation |  |  |  |  |  |  |
| No | 1,623 (25.06%) | 780 (25.18%) | 3,679.38 | 211.99 | REF | REF |
| Yes | 1,201 (18.54%) | 583 (18.82%) | 2,889.49 | 201.77 | 0.99 (0.89 - 1.11) | 0.86 (0.77 - 0.97) ^*^ |
| Other | 3,653 (56.40%) | 1,735 (56.00%) | 8,479.97 | 204.60 | 0.96 (0.89 - 1.05) | 0.97 (0.89 - 1.05) |
| Smoking status |  |  |  |  |  |  |
| No | 4,141 (63.93%) | 1,962 (63.33%) | 9,653.77 | 203.24 | REF | REF |
| Yes | 638 (9.85%) | 331 (10.68%) | 1,352.23 | 244.78 | 1.11 (0.99 - 1.25) | 1.09 (0.96 - 1.24) |
| Quit | 1,698 (26.22%) | 805 (25.98%) | 4,042.53 | 199.13 | 0.99 (0.92 - 1.08) | 1.03 (0.94 - 1.14) |
| Alcohol drinking |  |  |  |  |  |  |
| No | 4,829 (74.97%) | 2,286 (74.15%) | 11,209.84 | 203.93 | REF | REF |
| Yes | 584 (9.07%) | 291 (9.44%) | 1,450.44 | 200.63 | 1.01 (0.90 - 1.14) | 1.02 (0.90 - 1.16) |
| Quit | 1,028 (15.96%) | 506 (16.41%) | 2,373.49 | 213.19 | 1.05 (0.95 - 1.15) | 1.06 (0.96 - 1.18) |
| Betel consumption |  |  |  |  |  |  |
| No | 5,929 (91.62%) | 2,848 (91.99%) | 13,868.23 | 205.36 | REF | REF |
| Yes | 79 (1.22%) | 43 (1.39%) | 171.78 | 250.33 | 1.22 (0.90 - 1.65) | 1.12 (0.83 - 1.52) |
| Quit | 463 (7.15%) | 205 (6.62%) | 1,006.21 | 203.73 | 0.92 (0.80 - 1.06) | 0.89 (0.77 - 1.02) |
| Sports habits |  |  |  |  |  |  |
| No | 3,182 (49.12%) | 1,556 (50.23%) | 6,832.22 | 227.75 | REF | REF |
| Yes | 3,296 (50.88%) | 1,542 (49.77%) | 8,216.85 | 187.66 | 0.92 (0.86 - 0.99) ^*^ | 0.93 (0.87 - 1.00) ^*^ |
| Comorbidities |  |  |  |  |  |  |
| Diabetes mellitus |  |  |  |  |  |  |
| No | 3,735 (57.66%) | 1,581 (51.03%) | 9,737.06 | 162.37 | REF | REF |
| Yes | 2,743 (42.34%) | 1,517 (48.97%) | 5,312.01 | 285.58 | 1.44 (1.34 - 1.54) ^***^ | 1.47 (1.37 - 1.58) ^***^ |
| Hypertension |  |  |  |  |  |  |
| No | 1,649 (25.46%) | 713 (23.01%) | 3,997.63 | 178.36 | REF | REF |
| Yes | 4,829 (74.54%) | 2,385 (76.99%) | 11,051.43 | 215.81 | 1.20 (1.10 - 1.30) ^***^ | 1.23 (1.13 - 1.34) ^***^ |
| Gout |  |  |  |  |  |  |
| No | 5,223 (80.63%) | 2,516 (81.21%) | 11,884.56 | 211.70 | REF | REF |
| Yes | 1,255 (19.37%) | 582 (18.79%) | 3,164.51 | 183.92 | 0.92 (0.84 - 1.00) | 0.91 (0.83 - 1.00) |
| Heart failure |  |  |  |  |  |  |
| No | 6,276 (96.88%) | 3,011 (97.19%) | 14,644.23 | 205.61 | REF | REF |
| Yes | 202 (3.12%) | 87 (2.81%) | 404.83 | 214.90 | 0.92 (0.75 - 1.14) | 0.96 (0.78 - 1.19) |
| Heart disease |  |  |  |  |  |  |
| No | 6,232 (96.20%) | 2,982 (96.26%) | 14,442.25 | 206.48 | REF | REF |
| Yes | 246 (3.80%) | 116 (3.74%) | 606.81 | 191.16 | 0.96 (0.79 - 1.15) | 1.00 (0.83 - 1.21) |
| Cerebrovascular disease |  |  |  |  |  |  |
| No | 6,225 (96.09%) | 2,975 (96.03%) | 14,485.34 | 205.38 | REF | REF |
| Yes | 253 (3.91%) | 123 (3.97%) | 563.72 | 218.19 | 0.97 (0.81 - 1.17) | 1.01 (0.84 - 1.21) |
| Cirrhosis |  |  |  |  |  |  |
| No | 6,168 (95.21%) | 2,943 (95.00%) | 14,343.92 | 205.17 | REF | REF |
| Yes | 310 (4.79%) | 155 (5.00%) | 705.15 | 219.81 | 1.03 (0.88 - 1.21) | 1.00 (0.85 - 1.17) |
| Cancer |  |  |  |  |  |  |
| No | 5,919 (91.37%) | 2,859 (92.29%) | 13,881.24 | 205.96 | REF | REF |
| Yes | 559 (8.63%) | 239 (7.71%) | 1,167.83 | 204.65 | 0.89 (0.78 - 1.02) | 0.91 (0.80 - 1.04) |
| Tuberculosis |  |  |  |  |  |  |
| No | 6,474 (99.94%) | 3,097 (99.97%) | 15,042.44 | 205.88 | REF | REF |
| Yes | 4 (0.06%) | 1 (0.03%) | 6.62 | 150.99 | 0.47 (0.07 - 3.30) | 0.50 (0.07 - 3.58) |
| Hyperlipidemia |  |  |  |  |  |  |
| No | 4,999 (77.17%) | 2,329 (75.18%) | 11,435.20 | 203.67 | REF | REF |
| Yes | 1,479 (22.83%) | 769 (24.82%) | 3,613.87 | 212.79 | 1.09 (1.01 - 1.19) ^*^ | 1.08 (0.99 - 1.17) |
| Retina |  |  |  |  |  |  |
| No | 6,435 (99.34%) | 3,070 (99.10%) | 14,973.25 | 205.03 | REF | REF |
| Yes | 43 (0.66%) | 28 (0.90%) | 75.81 | 369.34 | 1.52 (1.05 - 2.20) ^*^ | 1.47 (1.01 - 2.13) ^*^ |
| Anemia |  |  |  |  |  |  |
| No | 5,773 (89.12%) | 2,754 (88.90%) | 13,513.11 | 203.80 | REF | REF |
| Yes | 705 (10.88%) | 344 (11.10%) | 1,535.96 | 223.96 | 1.02 (0.91 - 1.14) | 1.01 (0.90 - 1.13) |
| Immunity |  |  |  |  |  |  |
| No | 6,305 (97.33%) | 3,005 (97.00%) | 14,715.05 | 204.21 | REF | REF |
| Yes | 173 (2.67%) | 93 (3.00%) | 334.02 | 278.43 | 1.20 (0.97 - 1.47) | 1.11 (0.90 - 1.37) |
| Others |  |  |  |  |  |  |
| No | 5,779 (89.21%) | 2,772 (89.48%) | 13,266.03 | 208.96 | REF | REF |
| Yes | 699 (10.79%) | 326 (10.52%) | 1,783.03 | 182.84 | 0.92 (0.82 - 1.03) | 0.93 (0.83 - 1.05) |
| Medication habits |  |  |  |  |  |  |
| Prescription drugs |  |  |  |  |  |  |
| No | 329 (5.08%) | 149 (4.81%) | 907.30 | 164.22 | REF | REF |
| Yes | 6,149 (94.92%) | 2,949 (95.19%) | 14,141.76 | 208.53 | 1.09 (0.92 - 1.28) | 1.09 (0.93 - 1.29) |
| Chinese medicine |  |  |  |  |  |  |
| No | 5,821 (89.86%) | 2,801 (90.41%) | 13,419.53 | 208.73 | REF | REF |
| Yes | 657 (10.14%) | 297 (9.59%) | 1,629.54 | 182.26 | 0.94 (0.83 - 1.06) | 0.92 (0.82 - 1.04) |
| Chinese herb |  |  |  |  |  |  |
| No | 6,167 (95.20%) | 2,949 (95.19%) | 14,238.84 | 207.11 | REF | REF |
| Yes | 311 (4.80%) | 149 (4.81%) | 810.22 | 183.90 | 1.00 (0.85 - 1.18) | 1.00 (0.85 - 1.18) |
| Over-the-counter (OTC) drugs |  |  |  |  |  |  |
| No | 6,372 (98.36%) | 3,045 (98.29%) | 14,800.30 | 205.74 | REF | REF |
| Yes | 106 (1.64%) | 53 (1.71%) | 248.77 | 213.05 | 1.06 (0.81 - 1.39) | 1.06 (0.80 - 1.39) |
| Stimulating beverage |  |  |  |  |  |  |
| No | 6,477 (99.98%) | 3,098 (100.00%) | 15,047.06 | 205.89 | REF | REF |
| Yes | 1 (0.02%) | 0 (0.00%) | 2.01 | 0.00 | 0.00 (0.00 - 1.859E+54) | 0.00 (0.00 - 3.244E+54) |
| Folk prescription |  |  |  |  |  |  |
| No | 6,386 (98.58%) | 3,048 (98.39%) | 14,830.94 | 205.52 | REF | REF |
| Yes | 92 (1.42%) | 50 (1.61%) | 218.12 | 229.23 | 1.16 (0.88 - 1.53) | 1.18 (0.89 - 1.56) |
| Health foods |  |  |  |  |  |  |
| No | 6,208 (95.83%) | 2,968 (95.80%) | 14,462.46 | 205.22 | REF | REF |
| Yes | 270 (4.17%) | 130 (4.20%) | 586.60 | 221.62 | 1.04 (0.87 - 1.24) | 1.05 (0.88 - 1.25) |
| Oral pain killers |  |  |  |  |  |  |
| No | 6,187 (95.51%) | 2,970 (95.87%) | 14,272.63 | 208.09 | REF | REF |
| Yes | 291 (4.49%) | 128 (4.13%) | 776.43 | 164.86 | 0.86 (0.72 - 1.03) | 0.85 (0.71 - 1.01) |
| Intravenous pain killers |  |  |  |  |  |  |
| No | 6,392 (98.67%) | 3,059 (98.74%) | 14,844.94 | 206.06 | REF | REF |
| Yes | 86 (1.33%) | 39 (1.26%) | 204.13 | 191.06 | 0.88 (0.64 - 1.21) | 0.89 (0.65 - 1.22) |
| Unknown medication |  |  |  |  |  |  |
| No | 6,455 (99.64%) | 3,087 (99.64%) | 15,004.01 | 205.75 | REF | REF |
| Yes | 23 (0.36%) | 11 (0.36%) | 45.05 | 244.17 | 1.04 (0.57 - 1.88) | 1.08 (0.60 - 1.96) |
| Others |  |  |  |  |  |  |
| No | 6,352 (98.05%) | 3,033 (97.90%) | 14,789.40 | 205.08 | REF | REF |
| Yes | 126 (1.95%) | 65 (2.10%) | 259.66 | 250.32 | 1.11 (0.87 - 1.42) | 1.13 (0.88 - 1.44) |
| Medication usages |  |  |  |  |  |  |
| EPO |  |  |  |  |  |  |
| No | 4,176 (65.14%) | 1,783 (58.17%) | 10,252.37 | 173.91 | REF | REF |
| Yes | 2,235 (34.86%) | 1,282 (41.83%) | 4,743.30 | 270.28 | 1.48 (1.38 - 1.59) ^***^ | 1.46 (1.36 - 1.58) ^***^ |
| Active vitamin D |  |  |  |  |  |  |
| No | 5,669 (88.43%) | 2,711 (88.45%) | 12,929.93 | 209.67 | REF | REF |
| Yes | 742 (11.57%) | 354 (11.55%) | 2,065.73 | 171.37 | 0.95 (0.85 - 1.06) | 0.94 (0.84 - 1.05) |
| Pressure pills |  |  |  |  |  |  |
| No | 794 (12.38%) | 283 (9.23%) | 1,402.55 | 201.78 | REF | REF |
| Yes | 5,617 (87.62%) | 2,782 (90.77%) | 13,593.11 | 204.66 | 1.35 (1.19 - 1.52) ^***^ | 1.35 (1.19 - 1.52) ^***^ |
| ARB |  |  |  |  |  |  |
| No | 3,088 (48.17%) | 1,379 (44.99%) | 5,977.84 | 230.69 | REF | REF |
| Yes | 3,323 (51.83%) | 1,686 (55.01%) | 9,017.82 | 186.96 | 1.07 (1.00 - 1.15) | 1.07 (1.00 - 1.15) |
| ACEI |  |  |  |  |  |  |
| No | 5,807 (90.58%) | 2,739 (89.36%) | 13,114.77 | 208.85 | REF | REF |
| Yes | 604 (9.42%) | 326 (10.64%) | 1,880.89 | 173.32 | 1.12 (1.00 - 1.26) ^*^ | 1.14 (1.02 - 1.28) ^*^ |
| CCB |  |  |  |  |  |  |
| No | 2,421 (37.76%) | 1,026 (33.47%) | 5,050.93 | 203.13 | REF | REF |
| Yes | 3,990 (62.24%) | 2,039 (66.53%) | 9,944.73 | 205.03 | 1.20 (1.12 - 1.30) ^***^ | 1.19 (1.10 - 1.28) ^***^ |
| Diuretics |  |  |  |  |  |  |
| No | 3,473 (54.17%) | 1,439 (46.95%) | 8,294.06 | 173.50 | REF | REF |
| Yes | 2,938 (45.83%) | 1,626 (53.05%) | 6,701.60 | 242.63 | 1.40 (1.30 - 1.50) ^***^ | 1.46 (1.36 - 1.57) ^***^ |
| Iron supplement |  |  |  |  |  |  |
| No | 5,179 (80.78%) | 2,425 (79.12%) | 11,914.08 | 203.54 | REF | REF |
| Yes | 1,232 (19.22%) | 640 (20.88%) | 3,081.58 | 207.69 | 1.09 (1.00 - 1.19) | 1.10 (1.00 - 1.20) ^*^ |
| Calcium phosphate binders |  |  |  |  |  |  |
| No | 4,786 (74.65%) | 2,163 (70.57%) | 11,390.19 | 189.90 | REF | REF |
| Yes | 1,625 (25.35%) | 902 (29.43%) | 3,605.47 | 250.18 | 1.31 (1.21 - 1.41) ^***^ | 1.28 (1.18 - 1.38) ^***^ |
| Antidiabetic drugs |  |  |  |  |  |  |
| No | 3,756 (58.59%) | 1,617 (52.76%) | 9,239.48 | 175.01 | REF | REF |
| Yes | 2,655 (41.41%) | 1,448 (47.24%) | 5,756.18 | 251.56 | 1.32 (1.23 - 1.42) ^***^ | 1.33 (1.24 - 1.43) ^***^ |
| Lipid lowering drugs |  |  |  |  |  |  |
| No | 2,974 (46.39%) | 1,325 (43.23%) | 5,911.84 | 224.13 | REF | REF |
| Yes | 3,437 (53.61%) | 1,740 (56.77%) | 9,083.82 | 191.55 | 1.07 (1.00 - 1.15) | 1.04 (0.97 - 1.12) |
| Biochemical examination |  |  |  |  |  |  |
| SBP (mmHg), per SD increment | 133.31 ± 17.62 | 135.31 ± 18.41 |  |  | 1.18 (1.14 - 1.22)^***^ | 1.18 (1.14 - 1.23) ^***^ |
| DBP (mmHg), per SD increment | 74.34 ± 11.00 | 75.19 ± 11.33 |  |  | 1.11 (1.07 - 1.15) ^***^ | 1.09 (1.05 - 1.13) ^***^ |
| Hemoglobin (g/dL), per SD increment | 11.19 ± 2.19 | 10.96 ± 2.09 |  |  | 0.83 (0.80 - 0.87) ^***^ | 0.81 (0.77 - 0.84) ^***^ |
| Hematocrit (%), per SD increment | 26.08 ± 14.71 | 25.67 ± 14.54 |  |  | 0.94 (0.90 - 0.97) ^**^ | 0.94 (0.91 - 0.98) ^*^ |
| Urea Nitrogen (mg/dL), per SD increment | 41.46 ± 22.15 | 41.59 ± 20.71 |  |  | 1.09 (1.05 - 1.13) ^***^ | 1.08 (1.05 - 1.12) ^***^ |
| Uric acid (mg/dL), per SD increment | 7.77 ± 2.43 | 7.74 ± 2.10 |  |  | 1.00 (0.96 - 1.03) | 0.99 (0.96 - 1.03) |
| Albumin (g/dL), per SD increment | 3.99 ± 1.35 | 3.91 ± 1.87 |  |  | 0.59 (0.55 - 0.64) ^***^ | 0.59 (0.55 - 0.64) ^***^ |
| Cholesterol (mg/dL), per SD increment | 180.11 ± 47.78 | 183.72 ± 50.77 |  |  | 1.10 (1.07 - 1.14) ^***^ | 1.09 (1.05 - 1.14) ^***^ |
| Triglyceride (mg/dL), per SD increment | 156.73 ± 114.51 | 163.15 ± 124.47 |  |  | 1.07 (1.04 - 1.11) ^***^ | 1.06 (1.03 - 1.10) ^**^ |
| LDL (mg/dL), per SD increment | 101.85 ± 40.61 | 103.23 ± 41.46 |  |  | 1.03 (0.99 - 1.08) | 1.03 (0.98 - 1.07) |
| Fasting glucose (mg/dL), per SD increment | 118.13 ± 45.90 | 120.08 ± 48.56 |  |  | 1.05 (1.02 - 1.09) ^*^ | 1.06 (1.02 - 1.10) ^*^ |
| HbA1c (%), per SD increment | 6.78 ± 3.41 | 6.91 ± 4.27 |  |  | 1.06 (1.03 - 1.09) ^***^ | 1.06 (1.03 - 1.09) ^***^ |
| Urine PCR (mg/g), per SD increment | 1,629.12 ± 2,627.20 | 2,239.88 ± 3,037.90 |  |  | 1.28 (1.25 - 1.32) ^***^ | 1.28 (1.25 - 1.31) ^***^ |
| SD: standard deviation. Adjusted HR were considered age and sex for adjustments. ^***^p< 0.001; ^**^0.001<p<0.01; ^*^0.01<p<0.05  SDs were respectively 17.62 for SBP, 11 for DBP, 2.19 for hemoglobin, 14.71 for hematocrit, 22.15 for urea nitrogen, 2.43 for uric acid, 1.35 for albumin, 47.78 for cholesterol, 114.51 for triglyceride, 40.61 for LDL, 45.9 for fasting glucose, 3.41 for HbA1c, and 2627.2 for urine PCR | | | | | | |
