# Supplementary figures and images for: Long-Term Exposure to Fine Particulate Matter and the Deterioration of Estimated Glomerular Filtration Rate: A Cohort Study in Patients With Pre-End-Stage Renal Disease
Source: Front Public Health. 2022 Apr 8;10:858655. doi: 10.3389/fpubh.2022.858655 (PMC9024125; doi:10.3389/fpubh.2022.858655)

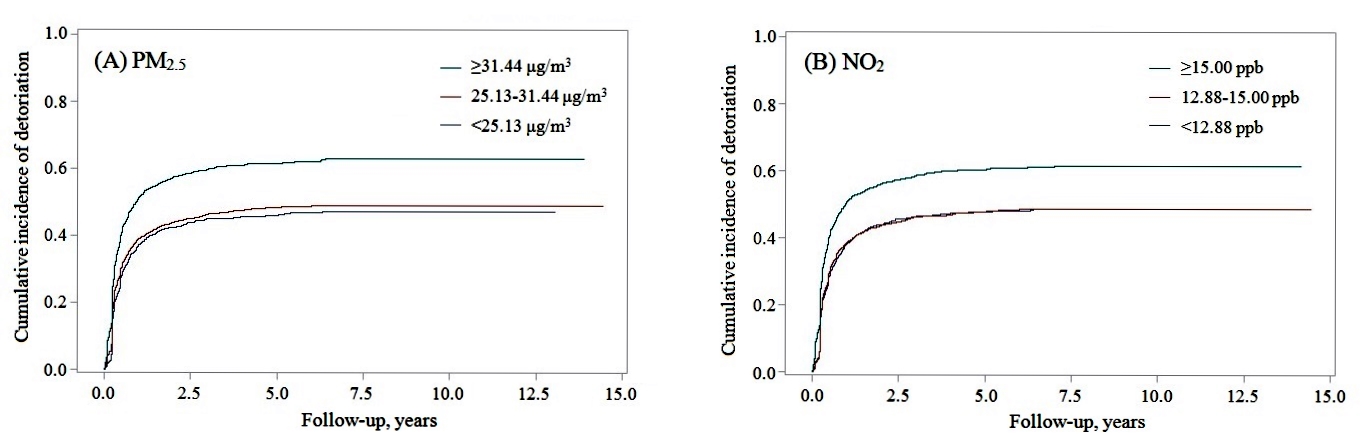

Supplement: Supplementary file 2 [file Image_1.JPEG]
